# Extended photoperiod improves growth and nutritional quality of pak choi under constant daily light integral

**DOI:** 10.3389/fpls.2025.1621513

**Published:** 2025-08-04

**Authors:** Kartika R. Susilo, Anastasia Eu, Britt Besemer, Ep Heuvelink, Ric C. H. de Vos, Leo F. M. Marcelis

**Affiliations:** ^1^ Department of Plant Sciences, Horticulture and Product Physiology Group, Wageningen University and Research, Wageningen, Netherlands; ^2^ Division of Urban Food Solutions, Department of Agri-tech and Food Innovation, Singapore Food Agency, Singapore, Singapore; ^3^ Business Unit Bioscience, Wageningen University and Research, Wageningen, Netherlands

**Keywords:** photoperiod, light intensity, pak choi, vitamin C, LUE, PSM, sole LED lights

## Abstract

**Introduction and Aim:**

The duration of light exposure each day, termed the photoperiod, is a crucial environmental cue that influence several aspects of plant physiology, including growth, development, and metabolic activity. Adjusting the photoperiod in controlled agriculture systems has the potential to improve crop yield and nutritional content. However, the benefits of longer photoperiods compared to higher light intensities under a fixed daily light integral (DLI) have not been thoroughly examined for many leafy vegetables. DLI is the total amount of light a plant receives per day and it is the product of photoperiod and light intensity. This study aimed to determine to what extent the effect of DLI on pak choi (*Brassica rapa L.* subsp. *chinensis*) growth, yield and quality depends on whether DLI is changed by light intensity (PPFD) or by photoperiod.

**Methods:**

Three cultivars (‘Hybrid Special’, ‘Red Summer’, and ‘Shanghai Green’) were grown under four different DLIs (10.8, 13.5, 16.2, and 18.9 mol m^-2^ d^-1^). These DLIs were achieved either by varying the photoperiod (12, 15, 18 and 21 hours) at a constant PPFD 250 µmol m^-2^ s^-1^ or by varying the PPFD (167, 208, 250, and 292 µmol m^-2^ s^-1^) at a constant photoperiod (18 hours).

**Results:**

Increasing DLI by extending the photoperiod resulted in more growth than increasing DLI by increasing PPFD. Photoperiod extension also generally resulted in higher light use efficiency and energy use efficiency than increasing DLI by increasing PPFD. The content of vitamin C, glucosinolates and many other metabolites increased significantly with higher DLI regardless whether DLI was increased through PPFD or photoperiod. However, DLI did not affect shelf life and overall visual quality.

**Discussion:**

These results suggest that extending photoperiod is a more effective strategy than increasing light intensity for optimizing leafy vegetable production in controlled environments.

## Introduction

Light is one of the main environmental factors that influence plant growth, development, and product quality. Light is pivotal in vertical farming, a plant production system in which crops are grown in vertically stacked layers under sole lamp light (Wong et al., 2020). Light is also a main factor determining capital and operational expenses of a vertical farm (Ahamed et al., 2023; Dutta Gupta and Agarwal, 2017), accounting for 70–80% of vertical farms’ electricity use (Kozai and Niu, 2020). The high energy requirement and resulting energy costs of vertical farming have hampered its development (Avgoustaki and Xydis, 2020; Kobayashi et al., 2022; Van Delden et al., 2021). LEDs are a good light source for vertical farms, due to their high energy efficiency and the versatility of the light spectrum (Cai et al., 2025; Pereira and Gomes, 2025). High energy costs make it essential to improve light use efficiency by adopting a good combination of photoperiod, photosynthetic photon flux density (PPFD), and light spectrum (Heuvelink et al., 2024; Pei et al., 2022). In addition to providing energy for photosynthesis, light acts as a signal that is mediated by photoreceptors (Brini et al., 2022). Through interactions with photoreceptors, cellular signaling and gene expression are influenced by light, which can affect biosynthesis and accumulation of specialized metabolites, also indicated as secondary metabolites (Van Brenk et al., 2024, 2025). These metabolites are found in most fruits and vegetables and are important for plant quality and protection against biotic and abiotic stress (Isah, 2019; Naing and Kim, 2021; Thoma et al., 2020; Wink, 1988).

One important way to assess and manage total light exposure in such systems is through the daily light integral (DLI), which is the product of PPFD and photoperiod, to calculate the total amount of light a growing area of plants receives per day. Plants grown at low DLI often show retarded growth. For example, lettuce (*Lactuca sativa* L.) grown at 3.7 mol m^-2^ d^-1^ was chlorotic and etiolated, which is typically the result of assimilate starvation (Paz et al., 2019). Within certain ranges DLI and plant growth have a positive linear relationship. Increasing DLI from 6.5 to 17 mol m^-2^ d^-1^ resulted in a linear increase in leaf fresh weight from 25 to 40 g per plant in hydroponic lettuce (Zhang et al., 2018). Moreover, an increase in DLI increased dry weight and leaf width of lettuce (Kelly et al., 2020; Pennisi et al., 2020b). However, Carotti et al. (2021) reported for lettuce that high DLI (23 and 43 mol m^-2^ d^-1^) caused a high rate of tipburn, a physiological disorder associate with local calcium deficiency (Barta and Tibbitts, 2000; Ertle and Kubota, 2023; Yuan et al., 2021).

The same DLI can be achieved by either utilizing a long photoperiod with low light intensity or a short photoperiod with high light intensity. However, plant growth and plant quality such as secondary metabolites can vary depending on the combination of PPFD and photoperiod, even with the same DLI. In lettuce, increasing the photoperiod from 12 to 21 hours increased shoot dry weight by 28 or 30% respectively, when DLI was maintained at 12 mol m^-2^ d^-1^ in a climate chamber (Elkins and van Iersel, 2020) or 17 mol m^-2^ d^-1^ under greenhouse conditions (Weaver and van Iersel, 2020). Similarly, Palmer and van Iersel (2020) found that extending the photoperiod from 10 to 20 hours at a constant DLI of 16 mol m^−2^ d^−1^ resulted in an 18% increase in dry weight of lettuce shoots. This increased growth with longer photoperiods, even with constant DLI, is likely due to higher photosynthetic efficiency under lower PPFD, the ability of a plant to convert absorbed light energy into chemical energy through photosynthesis.

Most research on vertical farming has so far focused on growing a small number of crops, such as lettuce or basil (*Ocimum basilicum*), while Asian leafy vegetables have received comparatively less attention (Boucher et al., 2023; Saapilin et al., 2022; Tan et al., 2020). This research uses pak choi (*Brassica rapa* L. subsp. *chinensis*) as a model plant of Asian leafy vegetables to study responses to light in vertical farming. Pak choi is an important crop that is widely cultivated and consumed in Asia and its consumption is also increasing in countries in Europe and elsewhere. Pak choi has a short growth cycle, high yield, and high nutritive value. It contains numerous health-benefiting compounds, such as glucosinolates, flavonoids, vitamins, and minerals (Jeon et al., 2018; Park et al., 2021). Although multiple studies have shown that increasing the DLI increases fresh mass, no study has demonstrated how different combinations of PPFD and photoperiod, while maintaining the same DLI affect the growth and quality attributes of pak choi. Therefore, the objective of this study is to determine to what extent the effect of DLI on pak choi growth, yield and quality depends on whether DLI is changed by light intensity or by photoperiod. We hypothesized that under the same DLI, a long photoperiod combined with low light intensity would increase biomass, leaf size, specific metabolite concentrations, and sugar content of pak choi more than a short photoperiod combined with high light intensity.

## Materials and methods

### Plant material and growth conditions

Two green-leaf cultivars, ‘Hybrid Special’ and ‘Shanghai Green’, and one red-leaf cultivar, ‘Red Summer’ of pak choi (seeds obtained from Ban Lee Huat Seed Pte Ltd, Singapore) were grown at a planting density of 44 plants per m^2^ in a climate chamber with a vertical farming setup consisting of eight compartments (1.3 × 0.8 × 1 m; L × W × H), each representing a different treatment (**Table 1**). ‘Hybrid Special’ and ‘Shanghai Green’ were chosen due to their widespread cultivation as a field crop in Asia, while ‘Red Summer’ was included as a representative red-leaf cultivar. One seed per plug was sown on stone wool trays (Grodan Rockwool B.V., The Netherlands). Seeds were kept in darkness for stratification (4°C, 96 h), then transferred to a climate room to germinate (Ilyas et al., 2022). Eleven days after sowing (DAS), morphologically similar plants were selected and transplanted into 10 × 10 × 6.5 cm stonewool blocks (Grodan Rockwool B.V., The Netherlands), with one plug per block. Each compartment contained 33 plants, consisting of nine plants per cultivar per treatment, along with two border plants per cultivar.

**Table 1:** Treatments creating different daily light integrals (DLI) by different photoperiods or PPFD.

PPFD (µmol m^-2^ s^-1^)	Photoperiod (h)	DLI (mol m^-2^ d^-1^)
DLI varied by PPFD		
167	18	10.8
208	18	13.5
250	18	16.2*
292	18	18.9
DLI varied by photoperiod		
250	12	10.8
250	15	13.5
250	18	16.2*
250	21	18.9
Highest DLI with the highest PPFD		
350	15	18.9

* The two identical treatments (DLI 16.2 mol m^-2^ d^-1^) are performed once but are shown twice for clarity.

Plants were grown under white light (GreenPower LED production module deep red/white 150, 2^nd^ generation; Philips, Eindhoven, The Netherlands). The total incident light intensity (μmol m^-2^ s^-1^ PAR) was measured at plant height using a PAR meter (LI-250A; Li-Cor Biosciences, Lincoln, NE, USA). The spectral photon composition was 9% (400–500 nm), 19% green-yellow (500–600 nm), 70% red (600–700 nm), and 2% FR (700–800 nm). Spectral composition was measured with a spectrometer (SS-110; Apogee Instruments, Logan, UT, USA). The [CO_2_] inside the climate chamber was set at 800 μmol mol^-1^. The daily temperature in the climate chamber was kept at 24°C ± 1°C, the relative humidity was 70% ± 10%. Two fans (12 V, 0.90 A) were installed per compartment, providing wind speeds of 0.2–0.3 m/s when off and 0.5–0.7 m/s when on, cycling on and off every 15 minutes to improve airflow. A nutrient solution (**Supplementary Table S1**) with a pH of 5.7 and an electrical conductivity (EC) of 2.3 dS m^-1^ was given every other day using an ebb-flow system.

### Light treatments

Different DLIs were achieved either by extending the photoperiod at the same light intensity or by increasing the light intensity while keeping the photoperiod constant (**Table 1**). Light treatments began at 11 DAS, with lights turned on at 04:00 for all treatments. While photoperiods differed between treatments (**Table 1**), the light duration was kept the same each day within each treatment. The experiment was conducted over three cultivation cycles, with treatments randomized across compartments for each cycle.

### Leaf gas exchange

To evaluate leaf photosynthesis rate, leaf gas exchange was measured 25–28 DAS, with measurements taken twice a day: 5 and 9 hours after the lights were turned on. Measurements were performed using an LI-6800XT photosynthesis system (LI-COR Biosciences Inc., USA) with a clear-top leaf chamber (LI-COR Part No.6800−40, area 6 cm^2^). Measurements were taken after the net photosynthesis rate (P_n_) had reached a steady state (∼4 min). During measurements, [CO_2_] was 800 μmol mol^-1^, leaf temperature was 24°C, relative humidity was 70%, and air flow rate through the system was 400 μmol s^-1^.

### Final harvest

A destructive harvest was conducted 32 DAS. Plant fresh weight and leaf area were measured for all nine plants per cultivar per treatment. Plant weight is defined as the total weight of all leaves; the short stem and roots (mostly inside the stonewool pot) were not measured. The fourth leaf (counted from the top of the plant) from each plant was frozen in liquid nitrogen immediately after measuring fresh weight and leaf area, then stored at -80°C for chemical analysis. The fifth leaf from each plant was removed, placed in individual zip-lock bags, and stored in a dark cold room (4°C) after fresh weight and leaf area measurements, to assess shelf-life. The remaining parts of the plants were dried in a ventilated oven at 105°C for 72 hours to determine dry weight. The specific leaf area (SLA) was calculated as the ratio between the total leaf area (cm²) and total leaf dry weight (g).

### Light use efficiency and electricity use efficiency

Light use efficiency (LUE) was calculated by dividing the final plant fresh weight by the total amount of PAR that was incident on the plant canopy over the entire growth period. Lighting energy use efficiency (EUE) was calculated by multiplying a fixed factor from LUE by an efficacy of 3.3 mol per MJ (for the LED lights) and a conversion factor of 3.6 MJ per kWh.

### Shelf-life and overall visual quality

Overall visual quality (OVQ) was assessed every 7 days during storage, up to 21 days post-harvest, using a scoring system based on visible symptoms of chilling injury. Scores ranged from 1 to 5, with specific criteria for each score (**Supplementary Table S2**). A score of 1 indicated severe deterioration, including extensive dark spots, significant color degradation, severe loss of turgor, wilting, and loss of leaf shininess. A score of 5 indicated excellent quality, with no visible signs of damage, vibrant color, firm turgor, and shiny leaves. A score of 3 was the threshold for consumer acceptability, marking the end of the shelf life. Scores were reduced for symptoms such as dark spots, color fading, loss of turgor, wilting, and diminished leaf shine.

### Total chlorophylls and carotenoid

Chlorophylls and carotenoids were measured using the method by López-Hidalgo et al. (2021). While still frozen, 1-mL 80% acetone was added to each sample. The resulting mixture was homogenized to facilitate the extraction of chlorophylls and carotenoids and then centrifuged at 21,300×*g*, 4°C, for 10 minutes. The supernatant was collected and 150 µl was added to a 96-well microplate. The absorbance was recorded at 664 nm, 649, and 470 nm for chlorophyll a, chlorophyll b, and carotenoids, respectively with 95% ethanol as a blank using the microplate spectrophotometer (SpectroMax iD3, USA). Chlorophyll a, chlorophyll b, and total carotenoid content (μg/ml) were calculated using the following formulas by Lichtenthaler (1987):


$$\text{Chl a }\left(\text{μg}/\text{ml}\right)=13.36{\text{A}}_{664}\;–\;5.19{\text{A}}_{649}$$

$$\text{Chl b }\left(\text{μg}/\text{ml}\right)=27.43{\text{A}}_{649}\;–\;8.12{\text{A}}_{664}$$

$$\text{Carotenoids }\left(\text{μg}/\text{ml}\right)=\frac{\left(1000{\text{A}}_{470}\right)\;–\left(2.13\text{ Chl a}\right)\;–\left(97.63\text{ Chl b}\right)}{209}$$

The concentrations of chlorophyll and carotenoid contents (μg/mL) were then converted based on the weight of the tissue extracted in 1 mL of the solution.

### Total antioxidant activity

The total antioxidant activity of plants was assessed from their 2,2-diphenyl-1-picrylhydrazyl (DPPH) radical scavenging ability, measured according to Brand-Williams et al. (1995) with some modifications, using a 96-wells microplates. Pak choi extracts were prepared by adding 1 ml of methanol to 40 mg of finely ground frozen leaf samples. The samples were centrifuged at 21,300×*g* for 10 minutes, then the supernatant was collected and incubated in the dark at room temperature for 30 minutes. Then, 12.5 µl of the leaf extract was added to 100 μl of a 0.1 mM DPPH radical solution in methanol and mixed directly in the 96-well plate. The control was created by adding 12.5 µl of methanol to the DPPH solution instead of the leaf extract. The absorbance was measured at 517 nm using the Spectramax iD3 reader (CA, USA). The scavenging ability of samples was calculated using the following formula:


$$\begin{array}{c} \text{DPPH radical scavenging activity }\left(\%\right)\\ =\frac{{\text{A}}_{517}\text{ control }-{\text{ A}}_{517}\text{ sample}}{{\text{A}}_{517}\text{ control}}\;\times 100 \end{array}$$

where A_517_ control is the absorbance of the control at 517 nm, and A_517_ sample is the absorbance of the sample at 517 nm.

### Total soluble sugar

Total soluble sugar content was analyzed as described by Savvides et al. (2014). A centrifuge tube containing 15 mg of freeze-dried plant material and 5 ml of 80% ethanol was briefly shaken and then placed in a water bath at 80°C for 20 minutes. The tubes were then centrifuged at 21,300×*g* for 5 minutes. A 1 ml aliquot of the supernatant was transferred to an Eppendorf tube and dried using a SpeedVac for 2 hour. Following drying, 1 ml of Milli-Q water was added to the tube, which was then shaken vigorously and placed in an ultrasonic bath (MH2800, Branson Ultrasonics, USA) for 10 minutes. Then the samples were shaken and centrifuged for an additional 10 minutes. A 20-fold dilution was prepared, and the samples were then analyzed with a High-Performance Liquid Chromatography (HPLC) Dionex system (GS 50 pump and PED 2 electrochemical detector). The total soluble sugars measured were the combined amounts of fructose, glucose, sucrose, and myo-inositol.

### Vitamin C

The reduced ascorbic acid in pak choi leaves was quantified by HPLC with UV detection at 260 nm (Bakir et al., 2023). In brief, 200 mg of powdered sample (fresh weight) was mixed with 1.2 mL of 5% metaphosphoric acid containing 1 mM DTPA as a redox metal chelator, centrifuged at 20,000×*g* for 20 min, and the supernatant was collected for analysis by HPLC using a YMC-Pack Pro C18 (150 × 4.6 mm) column (YMC, USA). To quantify vitamin C in the samples, an ascorbic acid calibration curve from 0.5 to 50 μg/ml was established by integrating the corresponding HPLC peak at 260 nm.

### LCMS-based metabolomics

HPLC sequentially coupled to light-absorbance detection and high resolution-mass spectrometry (LCMS) was applied as a comprehensive metabolomics approach, in order to determine effects of the light treatments on the global leaf metabolome of the pak choi plants. The metabolite extraction and analysis methods were principally as previously applied for LCMS-based metabolomics of various *Brassica* species (De Vos et al., 2011; Jeon et al., 2018). In brief, semi-polar compounds, including specialized compounds such as glucosinolates, flavonoids and other phenolic compounds, were extracted in 75% methanol (MeOH) acidified with 0.1% formic acid (FA). After sonication for 10 min and centrifugation for 15 min, 5 μl of the clear supernatant was injected by a Dionex UltiMate 3000 U-HPLC system (Thermo Scientific, USA). The compounds were separated on a Luna C18 column (2.0 x 150 mm, 3μm; Phenomenex) maintained at 40°C, using a 45-minute gradient of 5 to 75% acetonitrile acidified with 0.1% FA. Detection of eluting compounds was firstly with a photodiode array detector (200–700 nm) and secondly by a Q-Exactive Plus Orbitrap FTMS mass spectrometer (both Thermo Scientific, USA). Full scan MS data were generated with electrospray in positive/negative ionization switching mode at a mass resolution of 35,000 (FWHM) in the mass range of m/z 90 – 1,350.

### Targeted LCMS data processing for glucosinolates

Detected glucosinolates (GLS) were recognized in the LCMS profiles from their accurate negative ion masses ([M-H]^-^) and also from their indicative sulfate fragment ([M-H]^-^ = 96.9601). The peak areas of 11 manually identified GLS species were subsequently obtained using the QuanBrowser module of Xcalibur software (Thermo Scientific, USA), and the resulting table with relative peak intensities was used to compare the effects of light treatments.

### Untargeted LCMS data processing

The untargeted processing of the raw LCMS data files was conducted following the workflow developed by the Plant Metabolomics group of WPR-Bioscience, as described in Jeon et al. (2018) and Bakir et al. (2023). Chromatographic mass peaks above a noise threshold of 10,000 ions per scan were extracted and aligned using Metalign software (Lommen, 2009). The resulting peak lists were filtered to retain only signals present in at least 3 random samples and subsequently clustered into putative compounds using MSClust software (Tikunov et al., 2012). Data from positive and negative ionization modes were processed separately and then combined, resulting in a total of 2,056 putative compounds. A final filtering step retained only those compounds detected in all four biological replicates of at least one light treatment in at least one cultivar, resulting in a dataset of 1,165 (not yet annotated), metabolites. The most abundant masses in the ion-source mass spectrum of each compound were used to automatically search the KNApSAcK database (http://www.knapsackfamily.com/knapsack_core/top.php) for candidate metabolites previously detected in *Brassica* species. Per compound a maximum number of 3 candidate structures within 5 ppm deviation of observed mass from expected mass was retrieved (**Supplementary Table S3**).

### Statistical analysis

The experiment was conducted on three consecutive growth cycles, each constituting a complete experimental replicate (n = 3). These growth cycles were treated as blocks in the statistical analysis to account for environmental variation over time. For each treatment within a growth cycle, 12 plants were cultivated per cultivar, from which nine randomly selected plants were used for biomass, morphological and metabolomic measurements. These nine plants served as pseudo-replicates within each block.

For each cultivar, a one-way analysis of variance (ANOVA) was performed to assess the effect of DLI across eight light treatment combinations (resulting from different photoperiod and PPFD) on morphological, physiological, and some metabolomic traits. The block structure (growth cycle) was included in the model to control for temporal variability. Mean separation was conducted using Fisher’s protected least significant difference (LSD) test at a significance level of α = 0.05.

To further explore the joint effects of light quality and quantity, an analysis of covariance (ANCOVA) was performed, treating photoperiod and PPFD as qualitative factors, and DLI as a quantitative covariate. Interaction terms between DLI and treatment levels were included to assess differential slopes. This ANCOVA was performed on morphological, physiological, and some metabolomic traits.

All statistical analyses (ANOVA and ANCOVA) were performed using SAS version 9.4 (SAS Institute Inc, 2017). All corresponding regression equations are provided in **Supplementary Table S4**. Data visualization was conducted in R version 4.4.0 (R Core Team, 2024) using the ggplot2 package (Wickham, 2016) within RStudio (Posit Team, 2024).

Untargeted metabolomic data were analyzed using principal component analysis (PCA) and orthogonal partial least squares discriminant analysis (OPLS-DA), conducted in SIMCA version 17.0 (Sartorius AG, Germany). PCA analyses were performed to provide an overview of the metabolite data and to visualize groupings and trends related to cultivar differences and light treatments. In the second phase, OPLS-DA was conducted to separate systematic variation in the X-matrix into two components: one linearly related to the Y-matrix and one unrelated to it (Bylesjö, 2006). Prior to multivariate analysis, compound peak intensities were log10-transformed, mean-centered, and Pareto-scaled to normalize the data and ensure that all metabolites contributed equally regardless of units or magnitude. This standardization step was essential to account for differences in measurement scale among metabolites.

## Results

### Longer photoperiod outperforms higher PPFD in improving plant growth

**Figure 1:**
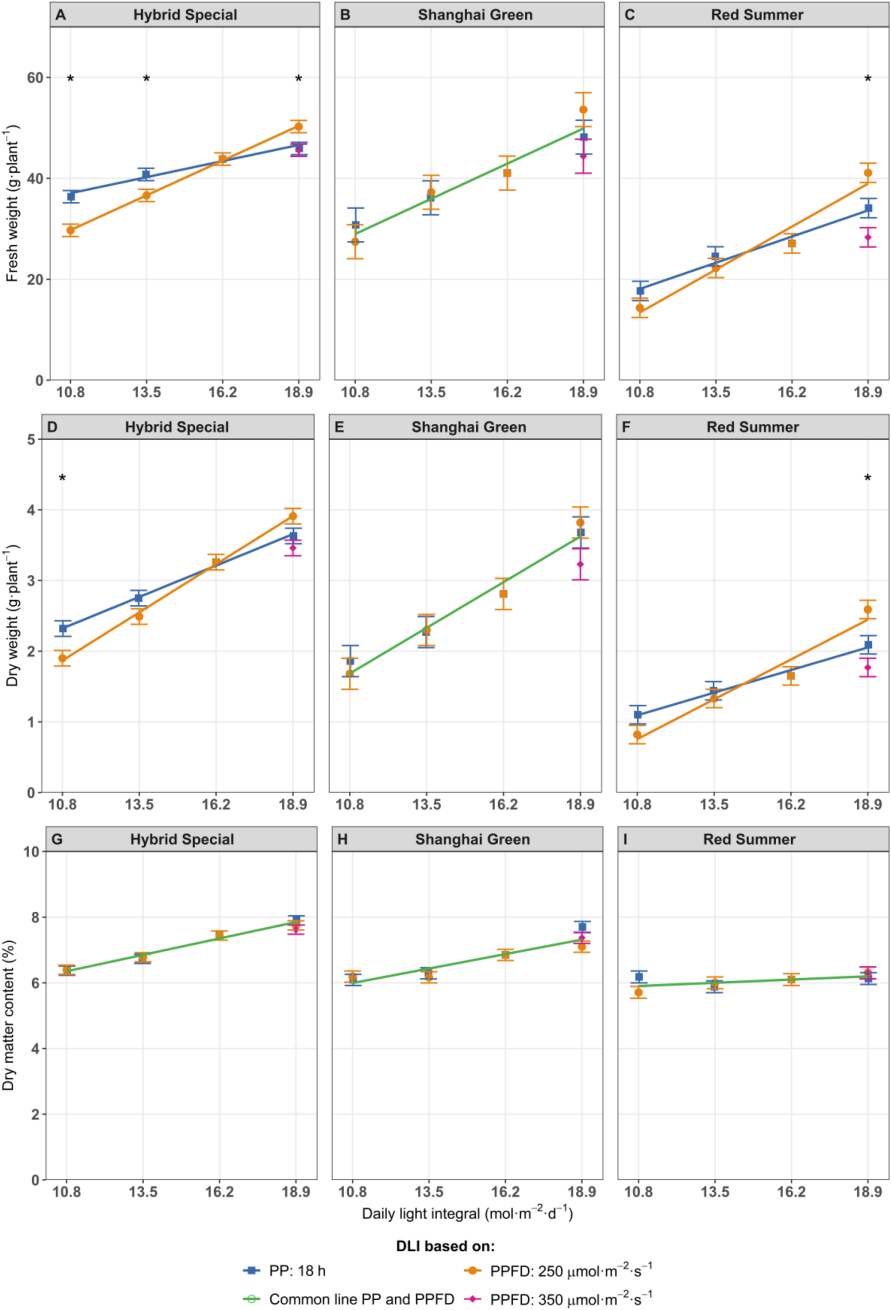
Fresh weight **(A–C)**, dry weight **(D–F)**, and dry matter content **(G–I)** of pak choi cultivars ‘Hybrid Special’ **(A, D, G)**, ‘Shanghai Green’ **(B, E, H)**, and ‘Red Summer’ **(C, F, I)** after four weeks of cultivation under red-blue-white LED light with eight different combinations of photoperiod and PPFD, resulting in four different daily light integrals (DLI). The DLI was increased by increasing PPFD (range 167–292 µmol m^-2^ s^-1^) at a constant photoperiod of 18 h (blue line) or by increasing photoperiod (range 12–21 hours) at a constant PPFD of 250 µmol m^-2^ s^-1^ (orange line). Purple diamond represents a third treatment at highest DLI, combining 350 µmol m^-2^ s^-1^ with 15 h photoperiod. Green line represents one common relationship with DLI, no difference between DLI increase obtained by increased PPFD or by increased photoperiod. Asterisks (*) indicate significant differences within the same DLI according to Fisher's protected LSD test at P = 0.05. Error bars represent the standard error of the mean.

Increasing DLI by extending the photoperiod from 12 h to 21 h at fixed PPFD resulted in higher fresh weights (**Figures 1A-C**) and dry weights (**Figures 1D-F**) than if DLI was increased by increasing light intensity with fixed photoperiod. Specifically, from the lowest to highest DLI, extending the photoperiod increased fresh weight by approximately 67%, compared to an increase of 26% with increasing PPFD under a fixed photoperiod. Furthermore, the treatment with the highest PPFD (350 µmol m^-^² s^-^¹) had the lowest fresh weight among treatments with the same DLI (18.9 mol m^-^² d^-^¹). Our results of higher fresh weight with longer photoperiods at the same DLI aligns with previous findings in several leafy vegetables. For example, when the photoperiod was extended from 12 to 16 hours, lettuce fresh weight and dry weight increased by 42% and 57% (Gavhane et al., 2023). Mizuna’s fresh weight increased by 19% when the photoperiod was extended from 10 to 24 hours (Palmer and van Iersel, 2020). Longer photoperiod also improves basil yield after cutting (Ciriello et al., 2023).

The dry matter content (DMC), defined as the ratio between plant dry weight and fresh weight, increased with higher DLI in the green-leaf cultivars (**Figures 1G, H**), irrespective of how DLI was increased (photoperiod or PPFD). Conversely, DLI did not affect DMC of ‘Red Summer’ (**Figure 1I**).

**Figure 2:**
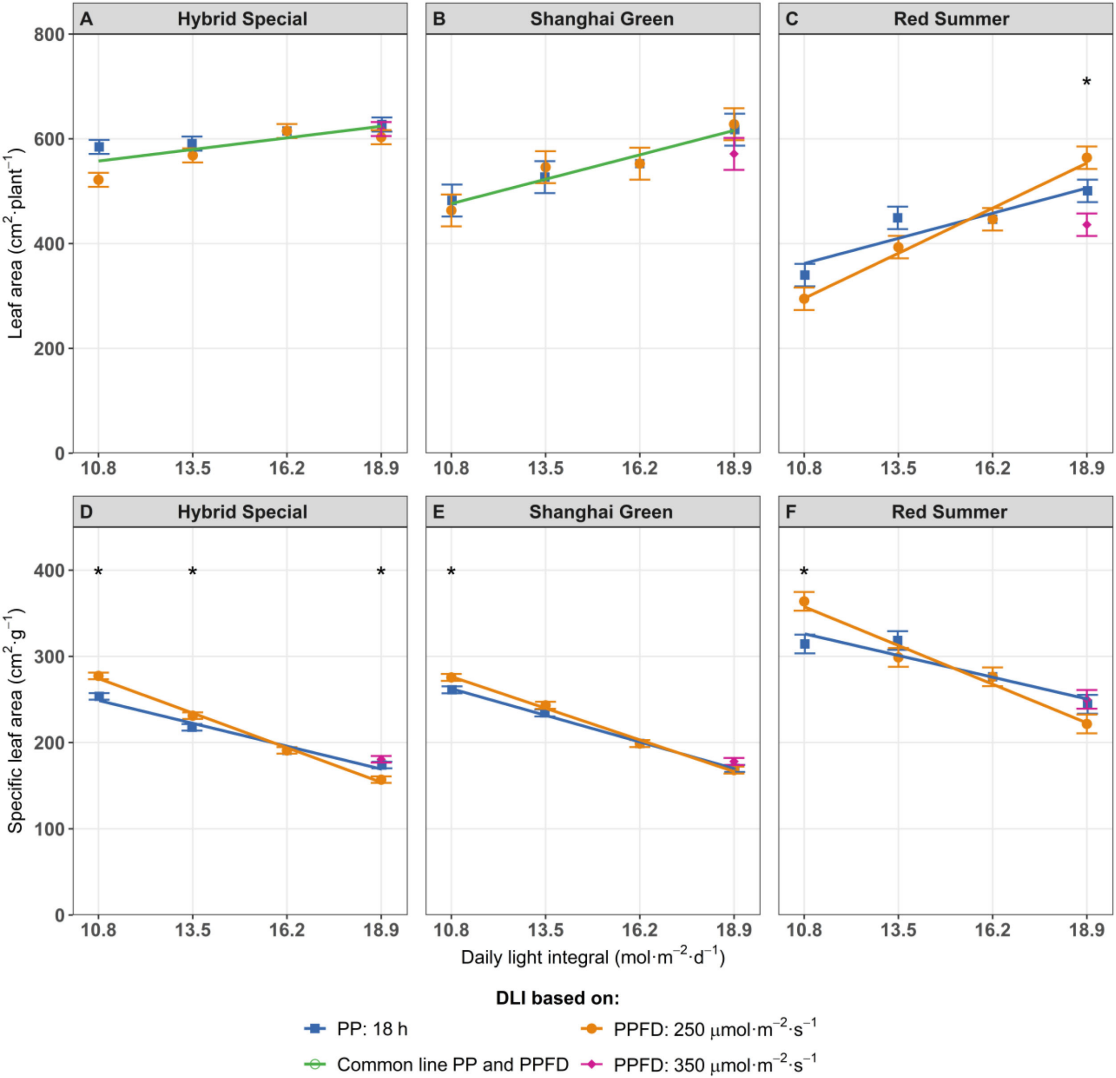
Leaf area **(A–C)**, and specific leaf area **(D–F)**, of pak choi cultivars ‘Hybrid Special’ **(A, D)**, ‘Shanghai Green’ **(B, E)**, and ‘Red Summer’ **(C, F)** after four weeks of cultivation under red-blue-white LED light with eight different combinations of photoperiod and PPFD, resulting in four different daily light integrals (DLI). The DLI was increased by increasing PPFD (range 167–292 µmol m^-2^ s^-1^) at a constant photoperiod of 18 h (blue line) or by increasing photoperiod (range 12–21 hours) at a constant PPFD of 250 µmol m^-2^ s^-1^ (orange line). Purple diamond represents a third treatment at highest DLI, combining 350 µmol m^-2^ s^-1^ with 15 h photoperiod. Green line represents one common relationship with DLI, no difference between DLI increase obtained by increased PPFD or by increased photoperiod. Asterisks (*) indicate significant differences within the same DLI according to Fisher's protected LSD test at P = 0.05.. Error bars represent the standard error of the mean.

In this research, the leaf area of green-leaf cultivars differed from the red-leaf cultivar in response to photoperiod and PPFD. In the green-leaf cultivars, increasing DLI by extending the photoperiod increased leaf area similarly as by increasing the PPFD (**Figures 2A, B**). However, for the red-leaf cultivar, increasing DLI by extending the photoperiod resulted in stronger increase in leaf area than by increasing PPFD (**Figure 2C**). Higher leaf area with an increase of PPFD from 260 to 290 μmol m^-^² s^-^¹ s(DLI from 16.8 to 18.8 mol m^-^² d^-^¹) was previously observed in lettuce (Kang et al., 2013). Similarly, basil showed increased leaf area when PPFD was more than 224 μmol m^-^² s^-^¹ (DLI 12.9 mol m^-^² d^-^¹) compared to PPFD 200 μmol m^-^² s^-^¹ (DLI 11.5 mol m^-^² d^-^¹) (Dou et al., 2020). Pennisi et al. (2020) also reported greater leaf area in lettuce and basil when increasing PPFD from 100 to 250 μmol m^-^² s^-^¹ (DLI from 5.8 to 14.4 mol m^-^² d^-^¹), although leaf area decreased at 300 μmol m^-^² s^-^¹ PPFD (DLI 17.3 mol m^-^² d^-^¹).

Specific leaf area (SLA) decreased with higher DLI, indicating thicker leaves at higher DLI (**Figures 2D–F**). Fukuda et al. (2004) also reported that longer photoperiods significantly decreased SLA in lettuce and tsukena (*Brassica campestris* L.) resulting from more palisade and spongy mesophyll. This decrease in SLA with increased DLI was stronger when DLI was increased by longer photoperiod, compared to when DLI was increased by higher PPFD. The combination of a long photoperiod and low PPFD resulted in higher leaf area and lower SLA compared to a short photoperiod and high PPFD at the same DLI, although these differences were only statistically significant at low DLI in ‘Red Summer’ and ‘Shanghai Green’.

### Light treatments affect photosynthesis, light use efficiency, and electricity use efficiency

**Figure 3:**
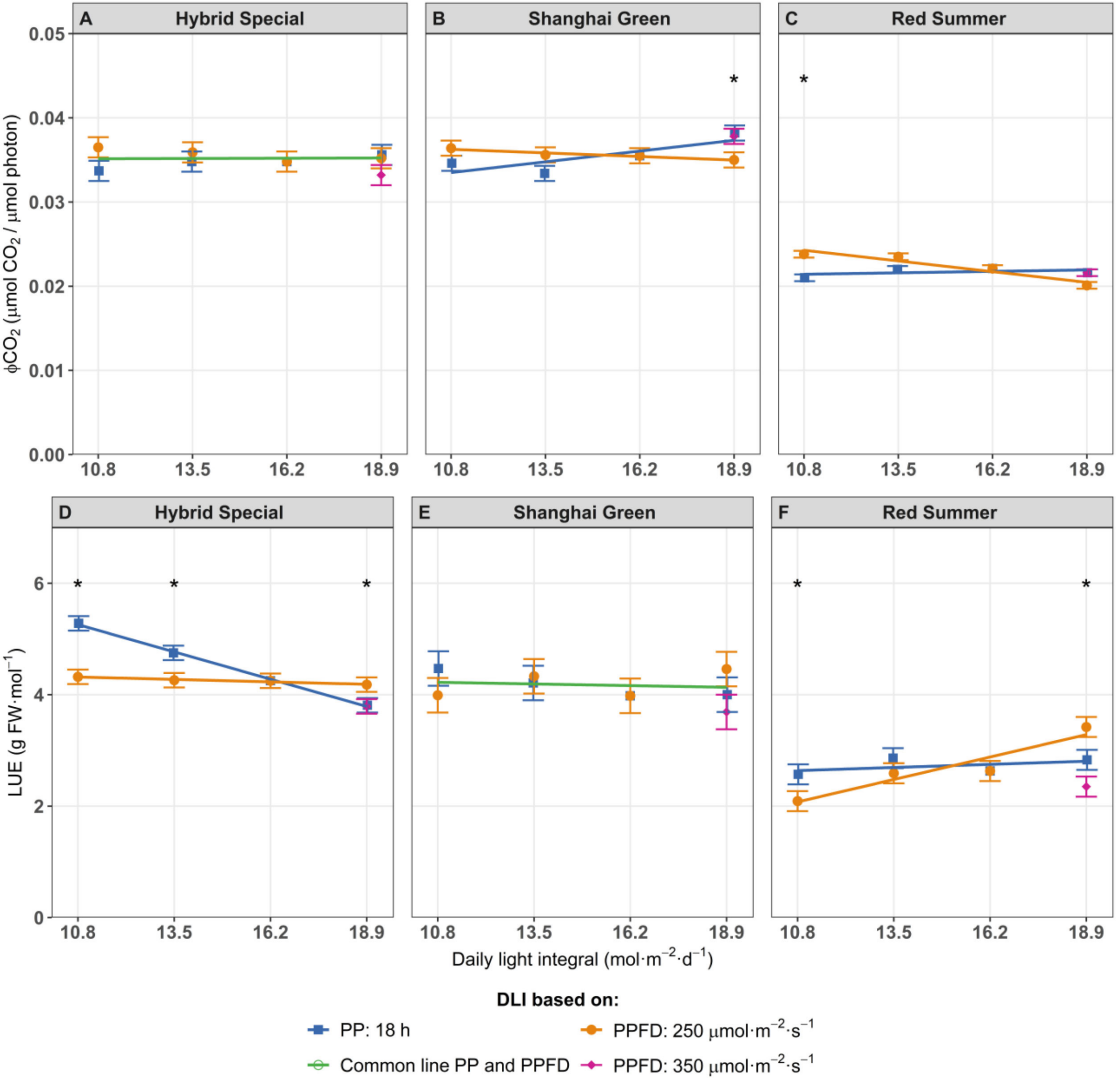
The quantum yield of CO_2_ assimilation (ΦCO_2_) **(A–C)**, and Light Use Efficiency of fresh weight **(D–F)**, of pak choi cultivars ‘Hybrid Special’ **(A, D)**, ‘Shanghai Green’ **(B, E)**, and ‘Red Summer’ **(C, F)** after four weeks of cultivation under red-blue-white LED light with eight different combinations of photoperiod and PPFD, resulting in four different daily light integrals (DLI). The DLI was increased by increasing PPFD (range 167–292 µmol m^-2^ s^-1^) at a constant photoperiod of 18 h (blue line) or by increasing photoperiod (range 12–21 hours) at a constant PPFD of 250 µmol m^-2^ s^-1^ (orange line). Purple diamond represents a third treatment at highest DLI, combining 350 µmol m^-2^ s^-1^ with 15 h photoperiod. Green line represents one common relationship with DLI, no difference between DLI increase obtained by increased PPFD or by increased photoperiod. Asterisks (*) indicate significant differences within the same DLI according to Fisher's protected LSD test at P = 0.05. Error bars represent the standard error of the mean.

For ‘Hybrid Special’, the quantum yield of CO_2_ assimilation (ΦCO_2_) was not affected by DLI (**Figure 3A**). For ‘Shanghai Green’, ΦCO_2_ increased with higher light intensity but slightly decreased with longer photoperiods (**Figure 3B**). ‘Red Summer’ showed a similar response as ‘Shanghai Green’, although the decline in ΦCO_2_ with increasing photoperiod was a bit stronger (**Figure 3C**).

LUE decreased when PPFD increased from 167 to 292 µmol m^-^² s^-^¹ at a fixed photoperiod for ‘Hybrid Special’ (**Figure 3D**), suggesting reduced photosynthetic efficiency at higher light intensities. This is consistent with previous findings (Jin et al., 2023; Powles, 2003), which showed higher PPFD reduced photosynthetic efficiency due to light saturation. In contrast, ‘Red Summer’ showed no change in LUE with increasing PPFD (**Figure 3F**). On the other hand, ‘Shanghai Green’ showed no significant changes in LUE across different photoperiods or PPFD levels, indicating its capacity to adapt to varying light conditions (**Figure 3E**). EUE mirrored LUE responses across all cultivars because the energy input is constant at the same DLI, so the effects on fresh weight similarly impact EUE (**Supplementary Figure S2**).

### DLI has no influence on overall visual quality

The number of plants showing tipburn at harvest increased with an increasing DLI (**Supplementary Figure S3**). Higher DLI has also been associated with increased sensitivity to tipburn in lettuce and spinach (Miao et al., 2023). Interestingly, tipburn appeared to be more frequent when DLI was increased by prolonged photoperiods compared to higher PPFD. Similar to lettuce (Birlanga et al., 2021; Ertle and Kubota, 2023), different cultivars of pak choi showed different degrees of tipburn sensitivity. ‘Hybrid Special’ showed the highest incidence of tipburn, within nearly all treatments one or more plants had tipburn. The overall visual quality of the leaves 21 days after harvesting was not affected by DLI during cultivation (**Supplementary Figure S4**).

### Effects of DLI on metabolite composition

#### Vitamin C, antioxidant radical scavenging activity and total soluble sugar

**Figure 4:**
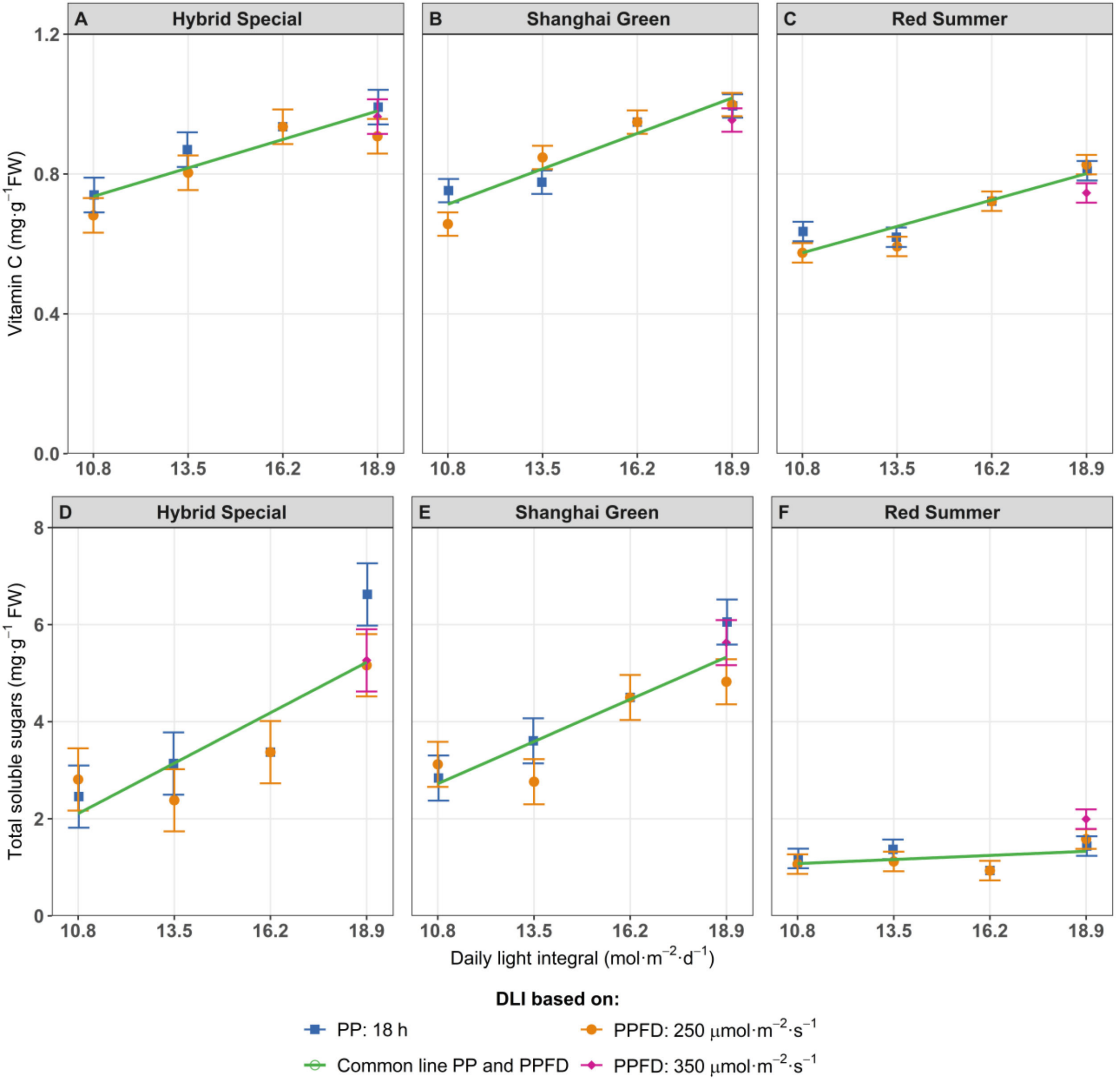
Vitamin C **(A–C)** and total soluble sugar **(D–F)** of pak choi cultivars ‘Hybrid Special’ **(A, D)**, ‘Shanghai Green’ **(B, E)**, and ‘Red Summer’ **(C, F)** after four weeks of cultivation under red-blue-white LED light with eight different combinations of photoperiod and PPFD, resulting four different daily light integrals (DLI). The DLI was increased by increasing PPFD (range 167–292 µmol m^-2^ s^-1^) at a constant photoperiod of 18 h (blue line) or by increasing photoperiod (range 12–21 hours) at a constant PPFD of 250 µmol m^-2^ s^-1^ (orange line). Purple diamond represents a third treatment at highest DLI, combining 350 µmol m^-2^ s^-1^ with 15 h photoperiod. Green line represents one common relationship with DLI, no difference between DLI increase obtained by increased PPFD or by increased photoperiod. Asterisks indicate significant differences within the same DLI according to Fisher’s protected LSD test at P = 0.05. Error bars represent the standard error of the mean.

In all three cultivars, vitamin C level increased consistently with increasing DLI. At the highest DLI (18.9 mol m^-2^ d^-1^), vitamin C content was approximately 25% higher compared to the lowest DLI (10.8 mol m^-2^ d^-1^) (**Figures 4A–C**). This increase was independent of how DLI was increased, whether through extended photoperiod or increased PPFD. Antioxidant capacity, measured by DPPH radical scavenging activity, also increased with DLI in ‘Hybrid Special’ and ‘Red Summer’ (**Supplementary Figure S5**). Similar to vitamin C, this trend was not affected by the method of DLI increase. However, in ‘Shanghai Green’, the antioxidant activity showed peak at DLI 16.2 mol m^-^² d^-^¹, suggesting an optimal rather than linear response. Total soluble sugar content showed a significant increase in response to higher DLI for both ‘Hybrid Special’ and ‘Shanghai Green’ (**Figures 4D, E**). A similar increase was observed for each of the four individual sugars (data not shown) and was consistent regardless of whether the DLI was increased via photoperiod extension or higher light intensity. In contrast, ‘Red Summer’ maintained consistently lower soluble sugar levels, which remained largely affected by changes in DLI (**Figure 4F**).

#### Chlorophylls, carotenoids, anthocyanins, and glucosinolates

Chlorophyll and carotenoid contents increased with higher DLI in all cultivars (**Supplementary Figures S6**, **S7**). This increase was independent of whether DLI was adjusted through photoperiod length or PPFD intensity.

**Figure 5:**
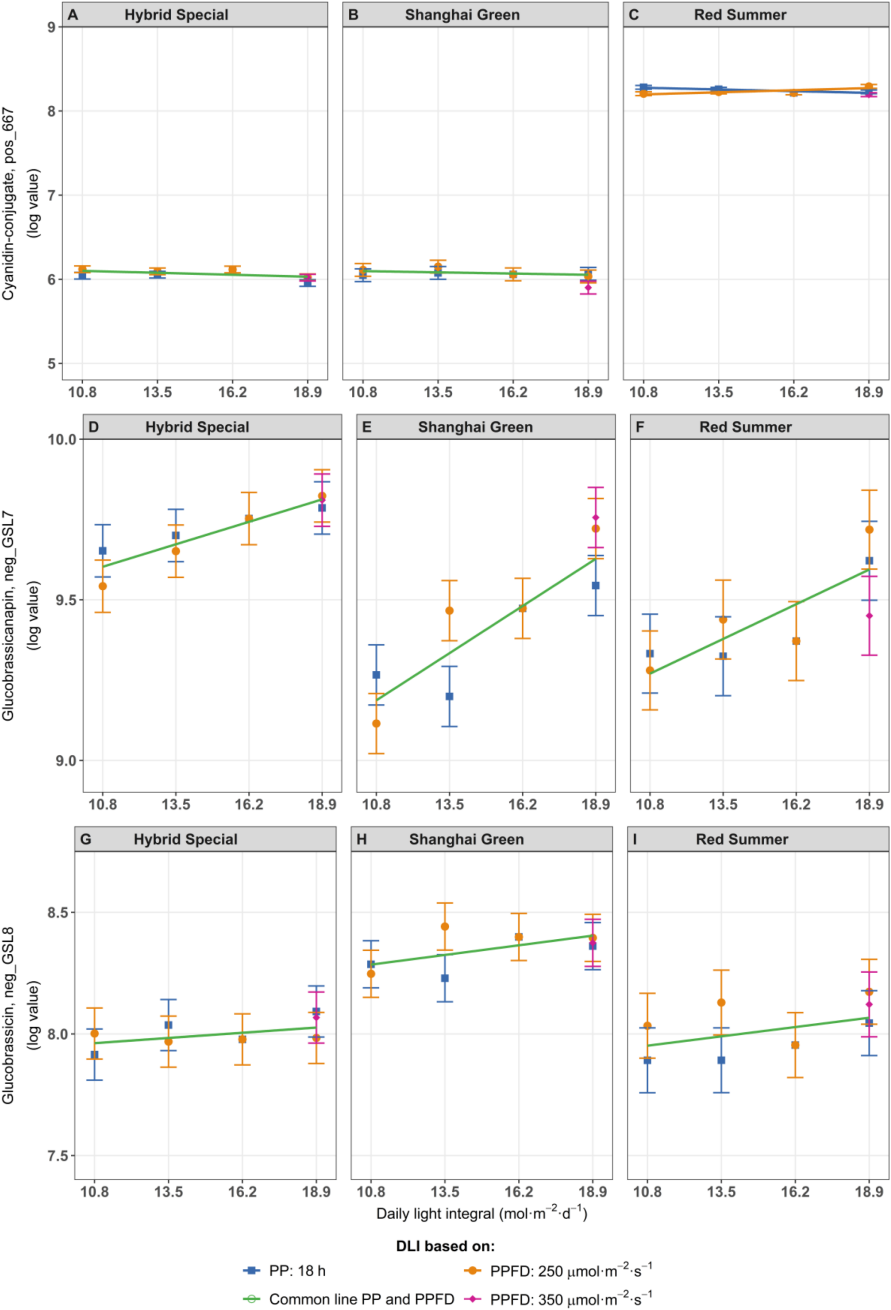
Log-transformed values of Cyanidin 3-(6-malonylglucoside)-feruloylglucoside-sinapoylglucoside (cyanidin-conjugate pos_667) **(A–C)**, glucobrassicanapin **(D–F)** and glucobrassicin **(G–I)** as main glucosinolates in pak choi cultivars ‘Hybrid Special’ **(A, D, G)**, ‘Shanghai Green’ **(B, E, H)**, and ‘Red Summer’ **(C, F, I)** after four weeks of cultivation under red-blue-white LED light with eight different combinations of photoperiod and PPFD, resulting in four different daily light integrals (DLI). The DLI was increased by increasing PPFD (range 167–292 µmol m^-2^ s^-1^) at a constant photoperiod of 18 h (blue line) or by increasing photoperiod (range 12–21 hours) at a constant PPFD of 250 µmol m^-2^ s^-1^ (orange line). Purple diamond represents a third treatment at highest DLI, combining 350 µmol m^-2^ s^-1^ with 15 h photoperiod. Green line represents one common relationship with DLI, no difference between DLI increase obtained by increased PPFD or by increased photoperiod. Asterisks indicate significant differences within the same DLI according to Fisher’s protected LSD test at P = 0.05. Error bars represent the standard error of the mean.

Five cyanidin-based anthocyanins were identified based on their specific absorbance at 500–520 nm and distinct accurate masses. These anthocyanins were primarily found in ‘Red Summer’, consistent with its reddish pigmentation. In this cultivar, anthocyanin levels (log-transformed) increased when DLI was raised by extending the photoperiod but decreased when DLI was increased by PPFD alone (**Figure 5C**, showing compound ID pos_667, identified as cyanidin 3-(6-malonylglucoside)-feruloylglucoside-sinapoylglucoside, as an example). Overall, a combination of a long photoperiod and low PPFD resulted in higher amounts of cyanidins compared to a short photoperiod and high PPFD at the same DLI, although the differences were not always statistically significant. In contrast, the green cultivars showed no significant changes in the levels of these compounds in response to increasing DLI (**Figures 5A, B**).

Regarding glucosinolates, glucobrassicanapin (alkenyl-type) and glucobrassicin (indole-type) are the main compound detected in pak choi (Kim et al., 2024). Glucobrassicanapin levels (log-transformed values) increased in all three cultivars with increasing DLI, regardless of whether the increase was due to photoperiod or PPFD (**Figures 5D–F**). In contrast, glucobrassicin levels was highest in ‘Shanghai Green’ but did not showed a consistent response to increase DLI across treatments or cultivars (**Figures 5G–I**).

### Untargeted LCMS-based metabolomics profiles

The untargeted LCMS-based metabolomics approach resulted in relative intensity values for a total of 1,602 compounds, including 11 targeted glucosinolates compounds and 1,591 untargeted compounds. These compounds were detected in at least one treatment or cultivar across the three biological replicates. The majority of these compounds represent semi-polar specialized metabolites (**Supplementary Table S3**).

**Figure 6:**
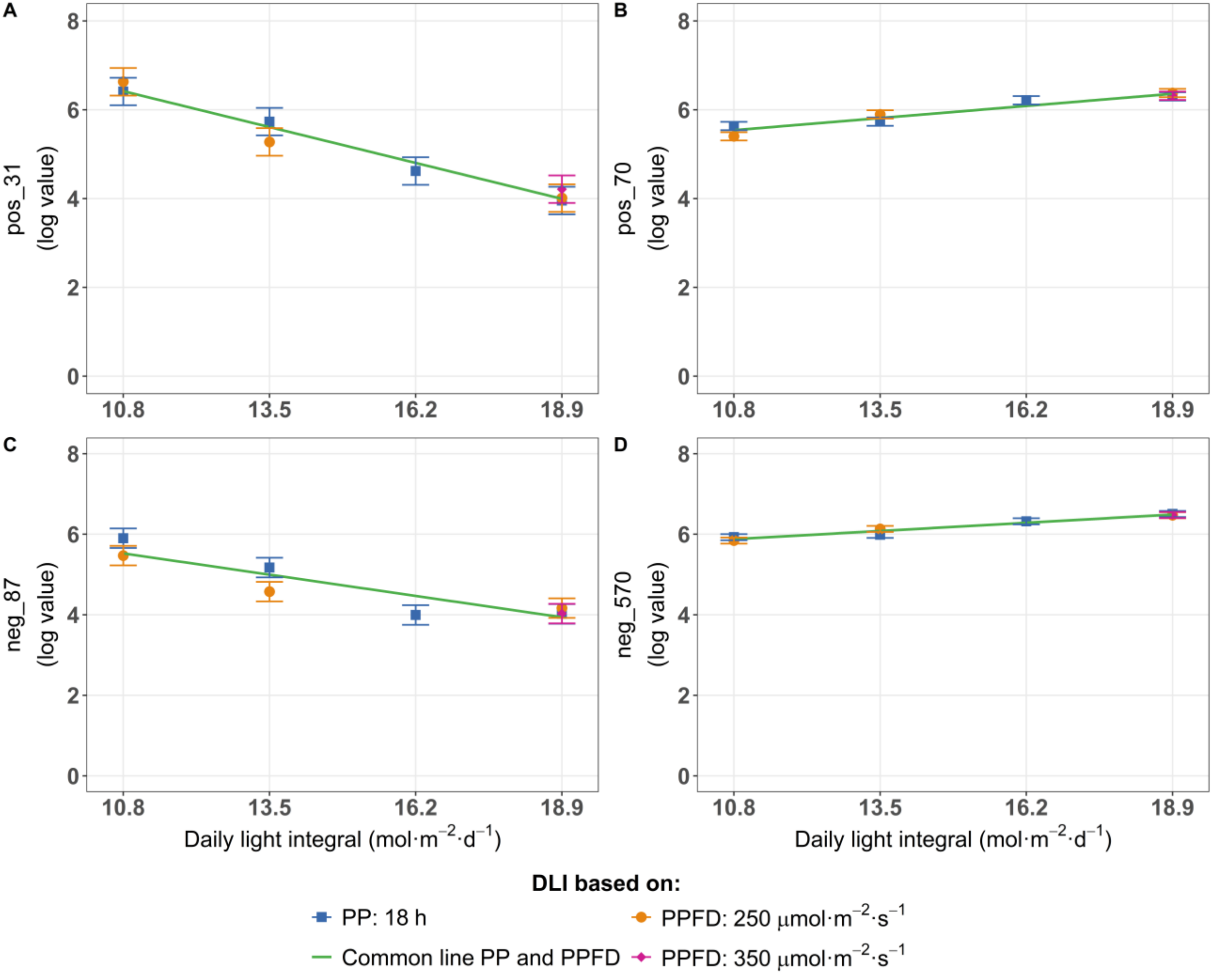
Log-transformed values of untargeted metabolites showing a negative correlation (< -0.8) across all three replicates **(A, C)** or a positive correlation (> 0.8) across all three replicates **(B, D)** with daily light integral (DLI) in pak choi ‘Shanghai Green’ determined after four weeks of cultivation under red-blue-white LED light. DLI either increased by increasing PPFD (range 167–292 µmol m^-2^ s^-1^) at a constant photoperiod of 18 h (blue symbol) or by increasing photoperiod (range 12–21 hours) at a constant PPFD of 250 µmol m^-2^ s^-1^ (orange symbol). Purple diamond represents a third treatment at highest DLI, combining 350 µmol m^-2^ s^-1^ with 15 h photoperiod. Green line connects average values for each DLI. Error bars represent the standard error of the mean.

Correlation coefficients were calculated to determine the relationship between DLI and the log-transformed value of untargeted metabolites. A positive correlation coefficient indicates that the compound intensity increased with higher DLI (**Figures 6A, C**), whereas a negative coefficient reflects a decrease as DLI increased (**Figures 6B, D**). Among 1,602 compounds, 45 showed a strong positive correlation (r > 0.95) while only 6 showed a strong negative correlation (r< -0.95), calculated across all cultivars in at least one replicate (**Supplementary Table S5**). Applying more stringent criteria revealed 16 compounds with a positive correlation coefficient (r > 0.8), with only 5 compounds having negative correlation (r< -0.8), present across all cultivars and in all three replicates (**Supplementary Table S6**). These compounds are considered robust and reproducible, making them promising candidates for further analysis.

**Figure 7:**
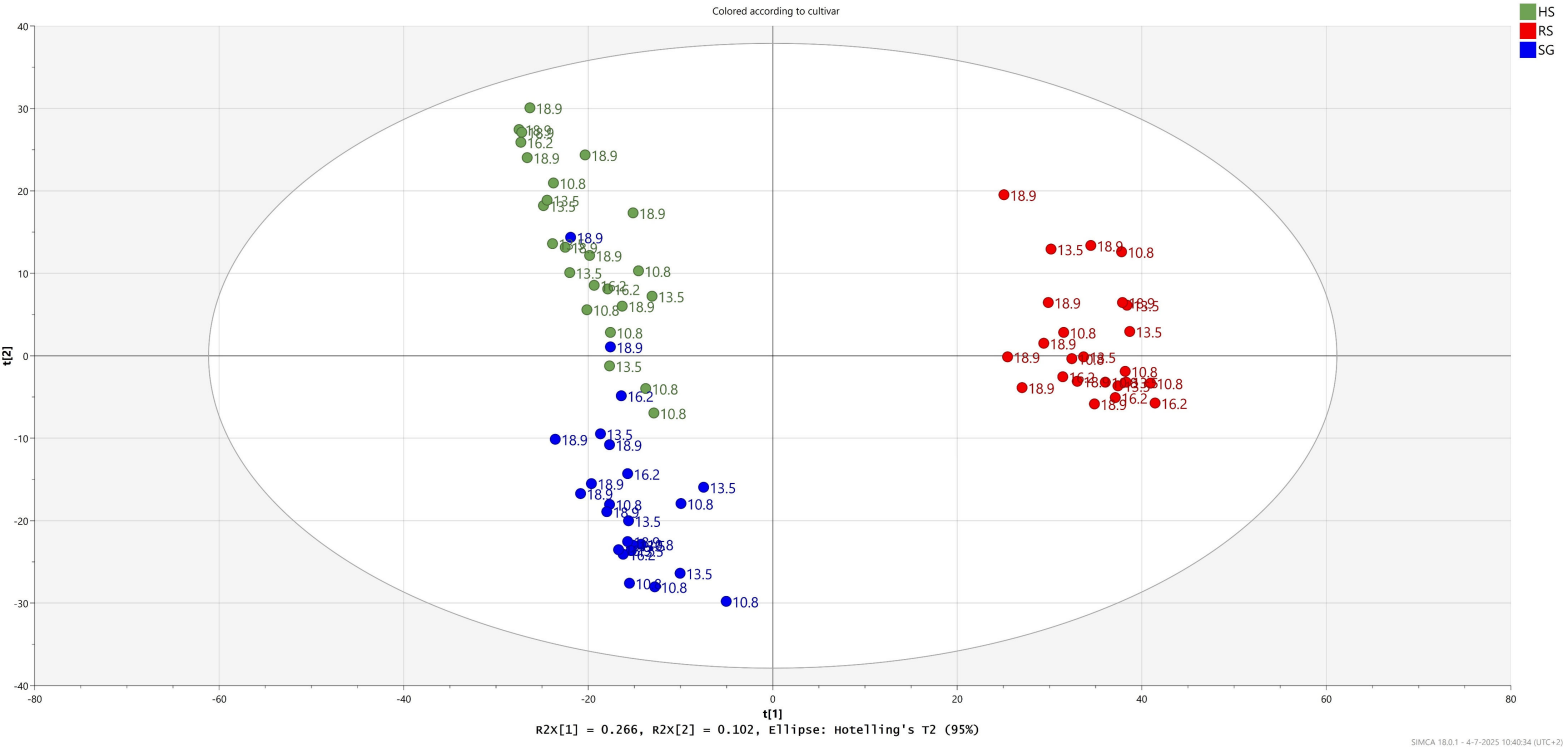
PCA of pak choi samples from three cultivars grown with increasing DLI intensity based on the variation in relative abundance of 1,591 compounds detected by the untargeted LCMS-based metabolomics approach. HS: ‘Hybrid Special’ (colored green). RS: ‘Red Summer’ (colored red) and ‘Shanghai Green’ (colored blue) after four weeks of cultivation under red-blue-white LED light with eight different combinations of photoperiod and PPFD, resulting four different daily light integrals (DLI; indicated in the sample codes).

Principal component analysis (PCA) was conducted to evaluate the overall effects of cultivar differences and light treatments on the metabolite profiles of pak choi. The first principal component (PC1) accounting for 26.6% of the total metabolite variation, separated samples based on cultivar. Specifically, ‘Red Summer’ was clearly distinguished from the two green-leaf cultivars, indicating substantial differences in metabolite composition (**Figure 7**). These differences likely include elevated anthocyanins content in ‘Red Summer’ (**Supplementary Table S3**). The second main component (PC2), explaining 10% of the total variation, further separated ‘Hybrid Special’ from ‘Shanghai Green’ (**Supplementary Figure S7**).

**Figure 8:**
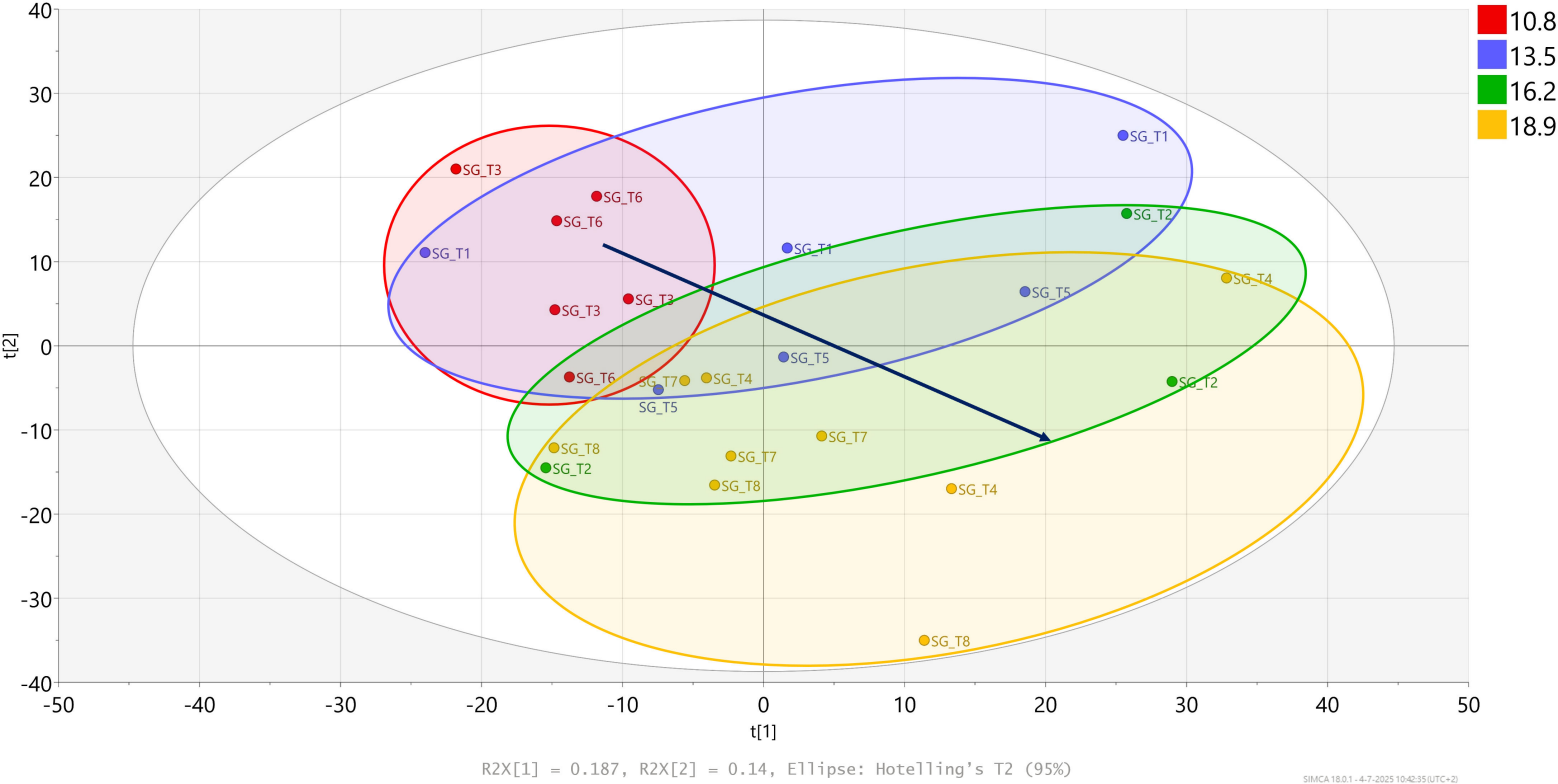
PCA of pak choi cultivar ‘Shanghai Green’ (SG) samples based on the variation in relative abundance of 1,591 compounds detected by the untargeted LCMS-based metabolomics approach. Plants were grown for four weeks under red-blue-white LED light at eight different combinations of photoperiod and light intensity, resulting four different daily light integrals (DLI). The circles enclose the four groups of samples exposed to the same DLI: 10.8 mol m^-2^ d^-1^ (red), 13.5 mol m^-2^ d^-1^ (brown), 16.2 mol m^-2^ d^-1^ (green) and 18.9 mol m^-2^ d^-1^ (yellow).

PCA analyses were also conducted for each cultivar separately to evaluate DLI effects on the global leaf metabolite profiles. A clear effect of light treatments on the overall metabolite profile was observed only on ‘Shanghai Green’ (**Figure 8**), while ‘Hybrid Special’ and ‘Red Summer’ showed no distinct separation based on DLI (**Supplementary Figures S8**, **S9**). To further investigate DLI effects in ‘Shanghai Green’, an orthogonal partial least squares discriminant analysis (OPLS-DA) was conducted to compare metabolites profiles between low DLI (10.8 mol m^-2^ d^-1^) and high DLI (18.9 mol m^-2^ d^-1^). This analysis revealed several DLI-responsive metabolites (**Supplementary Figure S10**). In particular, two phenylpropanoids, tentatively identified as feruloyl-glucoside and coumaroyl-quinic acid, were significantly upregulated under high DLI conditions (**Supplementary Figures S11A, B**). Conversely, a compound putatively identified as pyridoxal-5-phosphate (vitamin B6) was downregulated at high DLI (**Supplementary Figure S11C**). Interestingly, ‘Red Summer’ not only accumulated anthocyanins but also feruloyl-glucoside at high concentration. This result may indicate a constitutively upregulated phenylpropanoid/anthocyanidin pathway in this red-leaf cultivar as compared to both green-leaf cultivars.

## Discussion

### Effects of photoperiod extension on biomass accumulation and photosynthesis

Pak choi biomass increase responded differently depending on how DLI was increased, either by extending photoperiod or by increasing PPFD (**Figure 1**). When DLI was increased by extending photoperiod, fresh and dry biomass increase was larger (**Figure 1**). This aligns with observations in other species, such as lettuce, basil, kale, spinach, tomato, sweet pepper, strawberry, and arugula, suggesting that longer light periods at lower light intensities result in higher biomass production, even under a constant DLI (Carotti et al., 2021; Lanoue et al., 2022; Larsen et al., 2020; Mayorga-Gomez et al., 2024; Pennisi et al., 2020; Shibaeva et al., 2022; Urrestarazu et al., 2016; Zhang et al., 2018). One possible explanation lies in how plants manage light at different intensities. Under extended photoperiods with lower PPFD, photosynthesis can proceed more steadily without saturating the light response curve. In such conditions, photosystem II efficiency (ΦPSII) tends to be higher, supporting more efficient light conversion into chemical energy (Kelly et al., 2020). Distributing the same DLI over an extended photoperiod exposes plants to sub-saturating light for a longer duration, allowing for sustained carbon assimilation. Similar benefits have been observed in lettuce, where extended photoperiods improved daily electron transport rates at the same DLI (Elkins and van Iersel, 2020).

We initially hypothesized that, at the constant DLI, plant growth would be faster at lower PPFDs (and thus a longer photoperiods) due to the curvilinear light response of leaf photosynthesis. Although growth data supported the hypothesis, photosynthetic measurements did not entirely align with it. Gas exchange measurements revealed that quantum yield of CO_2_ fixation (ΦCO_2_) remained relatively stable across PPFD levels in ‘Hybrid Special’ and ‘Shanghai Green’ (**Figures 2A, B**), despite expectations that it would decline at higher PPFD. This constant ΦCO_2_ might reflect acclimation responses, possibly involving changes in leaf structure or pigment composition. One potential mechanism is a reduction in SLA, which results in thicker leaves, combined with an accumulation of photosynthetic pigments such as chlorophylls and carotenoids (Poorter et al., 2019). These traits may be associated with the relatively stable ΦCO_2_ observed at elevated PPFDs. Leaves appeared to acclimate to higher PPFD and DLI, maintaining a consistent ΦCO_2_ over a range of PPFD closer to saturation. This acclimation suggests that, while the curvilinear light-response behavior contributes to the observed growth patterns, additional factors such as leaf structural changes and pigment accumulation also play a role in maintaining photosynthetic performance at high PPFD. As PPFD approached saturation, a decrease in ΦCO_2_ might have been expected. However, the increase in leaf thickness may have counteracted this decline by enhancing photosynthetic capacity. The apparent balance between these opposing effects—decreased efficiency at high PPFD and increased capacity from leaf structural adaptation—could explain the relatively stable ΦCO_2_ we observed across treatments.

### Morphological adjustments: dry matter content and specific leaf area

Pak choi showed clear morphological adaptations to changes in DLI, particularly in terms of DMC and SLA, both of which reflect leaf structure and biomass partitioning. In green-leaf cultivars, DMC increased significantly with increasing DLI, regardless of whether DLI was increased by photoperiod extension or higher PPFD. In contrast, ‘Red Summer’ showed little change. Increases in DMC are often associated with improved postharvest quality and nutritional value, including higher carbohydrate and ascorbic acid concentrations (Kaiser et al., 2024; Min et al., 2021; Poorter et al., 2019).

Higher DLI provides more light energy for photosynthesis, resulting in increased sugar production. When carbon assimilation exceeds growth demand, excess carbohydrates are stored in tissues, contributing to higher DMC. This is typically reflected in thicker cell walls, denser cellular structures, and accumulation of storage compounds such as sugars and proteins (Gent, 2016; Poorter et al., 2009)). However, higher DMC doesn’t always equate to increased fresh weight. In our study, ‘Hybrid Special’ at constant PPFD dry weight increased from 1.9 to 3.9 g, and fresh weight increased from 30 to 50 g in the DLI range studied. However, if DMC could have been controlled, remaining constant at 6.3%, fresh weight would have increased from 30 to 62 g, and thus more than doubled.

SLA, an indicator of leaf thickness and density, declined with increasing DLI and longer photoperiods across all cultivars. Lower SLA indicates thicker leaves with higher biomass per unit area, which are typically associated to better stress tolerance and photosynthetic performance. Two meta-analysis including many studies and species by Poorter et al. (2009, 2019) revealed a nearly linear increase in leaf mass area (LMA, the inverse of SLA) with DLI increasing from 8 to 30 mol m^-2^ d^-1^. While thicker leaves may be considered a positive quality aspect, they also mean less leaf area produced from the same leaf biomass. This is particularly critical during early growth stages with a low leaf area index, where thicker leaves can lead to slower built-up of light interception, hence reducing crop photosynthesis and growth. In fast-growing crops like pak choi, lower SLA can slow canopy expansion and reduce light interception early in growth, which could limit total biomass accumulation.

To mitigate the effect of thicker leaves while maintaining a constant DMC, adjusting the growth temperature can be effective. Higher temperatures generally lead to thinner leaves and reduced DMC (Poorter et al., 2009). By adjusting temperature alongside changes in DLI, this may compensate for the increase in DMC we observed at higher DLI. Furthermore, maintaining an optimal source-to-sink ratio is essential for balanced plant growth. A higher DLI increases source-strength by augmenting the availability of assimilates, while higher temperatures increases sink-strength by accelerating metabolic processes and converting sugars into structural dry matter. Therefore, adjusting both light and temperature may help maintain this balance and optimize growth.

### Cultivar-specific responses and leaf pigmentation

The three cultivars responded differently to light treatments, with leaf anthocyanin content played a key role. The green-leaf cultivars, ‘Hybrid Special’ and ‘Shanghai Green’, maintained relatively stable quantum yield of CO_2_ fixation (ΦCO_2_) across all lighting conditions, whereas the red-leaf cultivar, ‘Red Summer’, exhibited a decline in ΦCO_2_ under extended photoperiods. This reduction is likely associated with higher anthocyanin levels in ‘Red Summer’. These compounds not only contribute to visual appeal by giving the leaves their red coloration, but also absorb excess light and provide photoprotection by shielding chloroplasts from damage, which can reduce the light available for photosynthesis (Gould et al., 2002; Landi et al., 2015). Despite the decrease in ΦCO_2_, ‘Red Summer’ maintained LUE under high PPFD, possibly due to the protective effects of anthocyanins in mitigating oxidative stress and photoinhibition (Liu and van Iersel, 2021; Palsha et al., 2024). This suggests that pigmentation influences how cultivars balance growth and stress responses under varying light conditions. Furthermore, longer photoperiods increased anthocyanin content in ‘Red Summer’. These pigments, mainly cyanidin derivatives, contribute to red coloration and respond strongly to light duration. Similar trends have been observed in red mustard, where anthocyanin accumulation peaked under a 16-hour photoperiod (Hwang et al., 2022), suggesting red-leaf cultivars may better tolerate prolonged light exposure.

Overall, the three cultivars showed broadly similar responses to the treatments. However, some differences were observed, particularly between the red- and green-leafed cultivars. Growers should take this into account when designing lighting strategies. In red-leafed cultivars, in particular, it may be more efficient to increase the DLI by extending the photoperiod rather than by increasing the PPFD.

### Effects of light intensity on light use efficiency

In ‘Hybrid Special,’ LUE dropped as PPFD increased from 167 to 292 µmol m^-^² s^-^¹, showing that the plants became less efficient under higher light intensity, likely due to light saturation (Jin et al., 2023; Powles, 2003). In contrast, ‘Red Summer’, showed no such decline, likely due to anthocyanins reducing light stress (**Figure 3F**). Meanwhile, ‘Shanghai Green’ maintained consistent LUE across all treatments, indicating a more flexible photosynthetic response. EUE followed a similar pattern to LUE because energy input was constant at each DLI. Thus, any gain in biomass directly translated to higher EUE. These findings emphasize that cultivars differ in how they manage light use and underline the need for cultivar-specific lighting strategies.

### Tipburn as a limiting factor under high DLI

Although higher DLI can enhance biomass production, it also increases the incidence of tipburn. particularly under extended photoperiods. All three cultivars showed more frequent tipburn under high DLI, especially when longer photoperiods were applied. Tipburn is a calcium-related disorder typically triggered by rapid leaf expansion, elevated transpiration, and high light demand. This disorder is worsened under rapid growth, increased transpiration, and higher light intensity, which collectively increase metabolic demand and impair nutrient transport (Barta and Tibbitts, 2000).

Mitigation strategies include maintaining high nighttime relative humidity (> 95%) to enhance calcium mobility to leaf margins (Vanhassel et al., 2015), and increasing airflow around the shoot meristem, which has been shown to prevent tipburn even under high PPFD, temperature, and CO_2_ conditions (Birlanga et al., 2021; Ertle and Kubota, 2023; Frantz et al., 2004). These prevention measures emphasize the importance of microclimate control (*i.e*., via adjustments to humidity, ventilation, and fertigation) to reduce calcium-related disorders in high-light production systems. Our findings suggest that although extended photoperiods can increase biomass, associated physiological stress like tipburn may reduce marketability, especially in dense vertical farming.

### Light conditions influence the composition of metabolites in pak choi leaves

DLI significantly influenced the composition of several metabolites in pak choi leaves. Our study showed that increasing DLI, either by increasing PPFD or extending the photoperiod, led to a significant increase in human health-related metabolites such as vitamin C (**Figures 4A–C**) and glucosinolates, including glucobrassicanapin and glucobrassicin (**Figures 5D–I**). Glucosinolates are sulfur-containing specialized metabolites known for their fungicidal, nematicidal, and bactericidal properties in plants (Mithen, 2001). These increases in health-related compounds suggest that optimizing light exposure could enhance the nutritional value of pak choi. In particular, ‘Shanghai Green’ showed significant changes in its overall leaf metabolome under higher DLI (**Supplementary Figure S10**). This cultivar had an increase of several phenolic compounds (**Figures 6A, C**), while others decreased (including a compound tentatively identified as vitamin B6) (**Figures 6B, D**). These analyses indicate that the effect of light conditions on the metabolome is cultivar-dependent and does not uniformly affect all metabolites – rather, photoperiod and PPFD may cause selective responses in specific, light-dependent metabolic pathways.

Our findings that light conditions influence the metabolome including phenolic and flavonoid compounds are consistent with similar observations in other crops, such as tomatoes (Ntagkas et al., 2019). Further studies are recommended to evaluate the nutritional implications of these DLI-related changes. Confirming compound identities through MSMS spectrometry and quantifying changes will be key for understanding the health value of pak choi under different lighting conditions.

Interestingly, the measurements of vitamin C in our samples more than doubled those reported for field-grown pak choi, which typically contains about 45 mg per 100 g fresh weight (Domínguez-Perles et al., 2014; USDA-ARS, 2018). This increase suggests a potential nutritional advantage of vertical farming. However, comparisons across studies should be made cautiously due to differences in cultivar, growth stage, and analytical methods. Future research should directly compare the metabolomes of field-grown and vertical-farming-grown plants from the same cultivars to better evaluate nutritional quality and to optimize light treatments without incurring excessive energy costs.

### Study limitations and directions for future research

This study examined increasing DLI through an altering either the duration of photoperiod or adjusting PPFD. Among these conditions, an 18-hour photoperiod emerged as a practical strategy to increase yield without causing excessive light stress, while still allowing for essential dark periods. However, alternative light strategies (*e.g.*, shorter photoperiods with higher PPFD or continuous photoperiod with low-intensity light) were not explored and may also prove effective as has been explore by (Gavhane et al., 2023; Y. Kang et al., 2024; Lanoue et al., 2022; Meng and Severin, 2024).

A notable limitation of this study is the absence of leaf temperature measurements. Although air temperature was controlled, radiant heat from LED lighting, especially at high PPFD, can raise leaf temperature independently of ambient conditions, potentially affecting photosynthesis and leaf development. Future experiments should include leaf temperature monitoring to separate the effects of light intensity from thermal influences.

While the cultivars studied are commercially relevant, including a broader genetic diversity would capture a wider range of responses. Molecular and transcriptomic analyses could further clarify the regulatory mechanisms behind morphological and metabolic changes. Finally, integrating physiological data with economic assessments will be crucial for optimizing lighting strategies in vertical farming.

## Conclusion

Increasing DLI resulted in higher fresh and dry weight of pak choi, which was stronger when DLI increased by extending photoperiod rather than increasing PPFD. Importantly, the improved yield of a higher DLI did not affect shelf-life. Furthermore, the content of vitamin C, total soluble sugar, and glucosinolates all increased with increasing DLI, regardless of how DLI was increased. Higher yields also occurred under longer photoperiods that had the same DLI, resulting in a higher LUE and EUE.

## Data Availability

The LC-MS data has been deposited in Zenodo and is available at the following DOI: https://doi.org/10.5281/zenodo.16271244.

## Supplementary material

Supplementary Figure 1 Photosynthesis rate of pak choi cultivars ‘Hybrid Special’ **(A)**, ‘Shanghai Green’ **(B)**, and ‘Red Summer’ **(C)** after four weeks of cultivation under red-blue-white LED light with eight different combinations of photoperiod and PPFD, resulting four different daily light integrals (DLI). The DLI was increased by increasing PPFD (range 167–292 µmol m^-2^ s^-1^) at a constant photoperiod of 18 h (blue line) or by increasing photoperiod (range 12–21 hours) at a constant PPFD of 250 µmol m^-2^ s^-1^ (orange line). Purple diamond represents a third treatment at highest DLI, combining 350 µmol m^-2^ s^-1^ with 15 h photoperiod. Green line represents one common relationship with DLI, not different between DLI increase obtained by increased PPFD or by increased photoperiod. Asterisks indicate significant differences within the same DLI according to Fisher’s protected LSD test at P = 0.05.

Supplementary Figure 2 Electricity Use Efficiency of pak choi cultivars ‘Hybrid Special’ **(A)**, ‘Shanghai Green’ **(B)**, and ‘Red Summer’ **(C)** after four weeks of cultivation under red-blue-white LED light with eight different combinations of photoperiod and PPFD, resulting four different daily light integrals (DLI). The DLI was increased by increasing PPFD (range 167–292 µmol m^-2^ s^-1^) at a constant photoperiod of 18 h (blue line) or by increasing photoperiod (range 12–21 hours) at a constant PPFD of 250 µmol m^-2^ s^-1^ (orange line). Purple diamond represents a third treatment at highest DLI, combining 350 µmol m^-2^ s^-1^ with 15 h photoperiod. Green line represents one common relationship with DLI, not different between DLI increase obtained by increased PPFD or by increased photoperiod. Asterisks indicate significant differences within the same DLI according to Fisher’s protected LSD test at P = 0.05.

Supplementary Figure 3 Percentage of tipburn of pak choi cultivars ‘Hybrid Special’ **(A)**, ‘Shanghai Green’ **(B)**, and ‘Red Summer’ **(C)** after four weeks of cultivation under red-blue-white LED light with eight different combinations of photoperiod and PPFD, resulting four different daily light integrals (DLI). The DLI was increased by increasing PPFD (range 167–292 µmol m^-2^ s^-1^) at a constant photoperiod of 18 h (blue line) or by increasing photoperiod (range 12–21 hours) at a constant PPFD of 250 µmol m^-2^ s^-1^ (orange line). Purple diamond represents a third treatment at highest DLI, combining 350 µmol m^-2^ s^-1^ with 15 h photoperiod. Green line represents one common relationship with DLI, not different between DLI increase obtained by increased PPFD or by increased photoperiod. Asterisks indicate significant differences within the same DLI according to Fisher’s protected LSD test at P = 0.05.

Supplementary Figure 4 Overall Visual Quality on day 21 of pak choi cultivars ‘Hybrid Special’ **(A)**, ‘Shanghai Green’ **(B)**, and ‘Red Summer’ **(C)** after four weeks of cultivation under red-blue-white LED light with eight different combinations of photoperiod and PPFD, resulting four different daily light integrals (DLI). The DLI was increased by increasing PPFD (range 167–292 µmol m^-2^ s^-1^) at a constant photoperiod of 18 h (blue line) or by increasing photoperiod (range 12–21 hours) at a constant PPFD of 250 µmol m^-2^ s^-1^ (orange line). Purple diamond represents a third treatment at highest DLI, combining 350 µmol m^-2^ s^-1^ with 15 h photoperiod. Green line represents one common relationship with DLI, not different between DLI increase obtained by increased PPFD or by increased photoperiod. Asterisks indicate significant differences within the same DLI according to Fisher’s protected LSD test at P = 0.05.

Supplementary Figure 5 Radical scavenging activity of pak choi cultivar ‘Hybrid Special’ **(A)**, ‘Shanghai Green’ **(B)**, and ‘Red Summer’ **(C)** after four weeks of cultivation under red-blue-white LED light with eight different combinations of photoperiod and PPFD, resulting four different daily light integrals (DLI). The DLI was increased by increasing PPFD (range 167–292 µmol m^-2^ s^-1^) at a constant photoperiod of 18 h (blue line) or by increasing photoperiod (range 12–21 hours) at a constant PPFD of 250 µmol m^-2^ s^-1^ (orange line). Purple diamond represents a third treatment at highest DLI, combining 350 µmol m^-2^ s^-1^ with 15 h photoperiod. Green line represents one common relationship with DLI, not different between DLI increase obtained by increased PPFD or by increased photoperiod. Asterisks indicate significant differences within the same DLI according to Fisher’s protected LSD test at P = 0.05.

Supplementary Figure 6 Total chlorophyll of pak choi cultivars ‘Hybrid Special’ **(A)**, ‘Shanghai Green’ **(B)**, and ‘Red Summer’ **(C)** after four weeks of cultivation under red-blue-white LED light with eight different combinations of photoperiod and PPFD, resulting four different daily light integrals (DLI). The DLI was increased by increasing PPFD (range 167–292 µmol m^-2^ s^-1^) at a constant photoperiod of 18 h (blue line) or by increasing photoperiod (range 12–21 hours) at a constant PPFD of 250 µmol m^-2^ s^-1^ (orange line). Purple diamond represents a third treatment at highest DLI, combining 350 µmol m^-2^ s^-1^ with 15 h photoperiod. Green line represents one common relationship with DLI, not different between DLI increase obtained by increased PPFD or by increased photoperiod. Asterisks indicate significant differences within the same DLI according to Fisher’s protected LSD test at P = 0.05.

Supplementary Figure 7 Carotenoid of pak choi cultivars ‘Hybrid Special’ **(A)**, ‘Shanghai Green’ **(B)**, and ‘Red Summer’ **(C)** after four weeks of cultivation under red-blue-white LED light with eight different combinations of photoperiod and PPFD, resulting four different daily light integrals (DLI). The DLI was increased by increasing PPFD (range 167–292 µmol m^-2^ s^-1^) at a constant photoperiod of 18 h (blue line) or by increasing photoperiod (range 12–21 hours) at a constant PPFD of 250 µmol m^-2^ s^-1^ (orange line). Purple diamond represents a third treatment at highest DLI, combining 350 µmol m^-2^ s^-1^ with 15 h photoperiod. Green line represents one common relationship with DLI, not different between DLI increase obtained by increased PPFD or by increased photoperiod. Asterisks indicate significant differences within the same DLI according to Fisher’s protected LSD test at P = 0.05.

Supplementary Figure 8 PCA of pak choi cultivar ‘Hybrid Special’ (HS) samples based on the variation in relative abundance of 1,591 compounds detected by the untargeted LCMS-based metabolomics approach. Plants were grown for four weeks under red-blue-white LED light with eight different combinations of photoperiod and light intensity, resulting four different daily light integrals (DLI). The circles enclose the four groups of samples exposed to the same DLI: 10.8 mol m^-2^ d^-1^ (red), 13.5 mol m^-2^ d^-1^ (brown), 16.2 mol m^-2^ d^-1^ (green) and 18.9 mol m^-2^ d^-1^ (yellow).

Supplementary Figure 9 PCA of pak choi cultivar ‘Red Summer’ (RS) samples based on the variation in relative abundance of 1,591 compounds detected by the untargeted LCMS-based metabolomics approach. Plants were grown for four weeks under red-blue-white LED light with eight different combinations of photoperiod and light intensity, resulting four different daily light integrals (DLI). The circles enclose the four groups of samples exposed to the same DLI: 10.8 mol m^-2^ d^-1^ (red), 13.5 mol m^-2^ d^-1^ (brown), 16.2 mol m^-2^ d^-1^ (green) and 18.9 mol m^-2^ d^-1^ (yellow).

Supplementary Figure 10 Supervised OPLS-DA plots of LC-MS data (+) for ‘Shanghai Green’ pak choi (SG) samples showed a separation between low DLI treatments (10.8 mol m^-^² d^-^¹, marked in red) and high DLI treatments (18.9 mol m^-^² d^-^¹, marked in yellow), indicating significant metabolic differences. Low DLI conditions include treatments T3 (PP 18 h, PPFD 167 µmol m^-^² s^-^¹) and T6 (PP 12 h, PPFD 250 µmol m^-^² s^-^¹), while high DLI include treatments T4 (PP 18 h, PPFD 292 µmol m^-^² s^-^¹), T7 (PP 21 h, PPFD 250 µmol m^-^² s^-^¹), and T8 (PP 15 h, PPFD 350 µmol m^-^² s^-^¹).

Supplementary Figure 11 Log-transformed values of untargeted metabolites across all three biological replicates in pak choi cultivar ‘Shanghai Green’ as a function of daily light integral (DLI) determined after four weeks of cultivation under red-blue-white LED light. DLI either increased by increasing PPFD (range 167–292 µmol m^-2^ s^-1^) at a constant photoperiod of 18 h (blue symbol) or by increasing photoperiod (range 12–21 hours) at a constant PPFD of 250 µmol m^-2^ s^-1^ (orange symbol). Purple diamond represents a third treatment at highest DLI, combining 350 µmol m^-2^ s^-1^ with 15 h photoperiod. Green line connects average values for each DLI.
