# Supplementary material for: Extended photoperiod improves growth and nutritional quality of pak choi under constant daily light integral
Source: Front Plant Sci. 2025 Aug 4;16:1621513. doi: 10.3389/fpls.2025.1621513 (PMC12358294; doi:10.3389/fpls.2025.1621513)
Supplement: Supplementary file 1 [file DataSheet1.docx]

**Extended Photoperiod Improves Growth and Nutritional Quality of Pak Choi Under Constant Daily Light Integral**

Kartika R. Susilo^1^, Anastasia Eu^2^, Britt Besemer^1^, Ep Heuvelink^1*^, Ric de Vos^1^, and Leo F. M. Marcelis^1^

^1^Wageningen University and Research, The Netherlands

^2^Singapore Food Agency, Singapore

**SUPPLEMENTARY DATA**





***Figure S1.*** *Photosynthesis rate of pak choi cultivars ‘Hybrid Special’ (A), ‘Shanghai Green’ (B), and ‘Red Summer’ (C) after four weeks of cultivation under red-blue-white LED light with eight different combinations of photoperiod and PPFD, resulting four different daily light integrals (DLI). The DLI was increased by increasing PPFD (range 167 – 292 µmol m^-2^ s^-1^) at a constant photoperiod of 18 h (blue line) or by increasing photoperiod (range 12 – 21 hours) at a constant PPFD of 250 µmol m^-2^ s^-1^ (orange line). Purple diamond represents a third treatment at highest DLI, combining 350 µmol m^-2^ s^-1^ with 15 h photoperiod. Green line represents one common relationship with DLI, not different between DLI increase obtained by increased PPFD or by increased photoperiod. Asterisks indicate significant differences within the same DLI according to Fisher's protected LSD test at P = 0.05.*





***Figure S2.*** *Electricity Use Efficiency of pak choi cultivars ‘Hybrid Special’ (A), ‘Shanghai Green’ (B), and ‘Red Summer’ (C) after four weeks of cultivation under red-blue-white LED light with eight different combinations of photoperiod and PPFD, resulting four different daily light integrals (DLI). The DLI was increased by increasing PPFD (range 167 – 292 µmol m^-2^ s^-1^) at a constant photoperiod of 18 h (blue line) or by increasing photoperiod (range 12 – 21 hours) at a constant PPFD of 250 µmol m^-2^ s^-1^ (orange line). Purple diamond represents a third treatment at highest DLI, combining 350 µmol m^-2^ s^-1^ with 15 h photoperiod. Green line represents one common relationship with DLI, not different between DLI increase obtained by increased PPFD or by increased photoperiod. Asterisks indicate significant differences within the same DLI according to Fisher's protected LSD test at P = 0.05.*





***Figure S3.*** *Percentage of tipburn of pak choi cultivars ‘Hybrid Special’ (A), ‘Shanghai Green’ (B), and ‘Red Summer’ (C) after four weeks of cultivation under red-blue-white LED light with eight different combinations of photoperiod and PPFD, resulting four different daily light integrals (DLI). The DLI was increased by increasing PPFD (range 167 – 292 µmol m^-2^ s^-1^) at a constant photoperiod of 18 h (blue line) or by increasing photoperiod (range 12 – 21 hours) at a constant PPFD of 250 µmol m^-2^ s^-1^ (orange line). Purple diamond represents a third treatment at highest DLI, combining 350 µmol m^-2^ s^-1^ with 15 h photoperiod. Green line represents one common relationship with DLI, not different between DLI increase obtained by increased PPFD or by increased photoperiod. Asterisks indicate significant differences within the same DLI according to Fisher's protected LSD test at P = 0.05.*





***Figure S4.*** *Overall Visual Quality on day 21 of pak choi cultivars ‘Hybrid Special’ (A), ‘Shanghai Green’ (B), and ‘Red Summer’ (C) after four weeks of cultivation under red-blue-white LED light with eight different combinations of photoperiod and PPFD, resulting four different daily light integrals (DLI). The DLI was increased by increasing PPFD (range 167 – 292 µmol m^-2^ s^-1^) at a constant photoperiod of 18 h (blue line) or by increasing photoperiod (range 12 – 21 hours) at a constant PPFD of 250 µmol m^-2^ s^-1^ (orange line). Purple diamond represents a third treatment at highest DLI, combining 350 µmol m^-2^ s^-1^ with 15 h photoperiod. Green line represents one common relationship with DLI, not different between DLI increase obtained by increased PPFD or by increased photoperiod. Asterisks indicate significant differences within the same DLI according to Fisher's protected LSD test at P = 0.05.*

**

**

***Figure S5.*** *Radical scavenging activity of pak choi cultivar ‘Hybrid Special’ (A), ‘Shanghai Green’ (B), and ‘Red Summer’ (C) after four weeks of cultivation under red-blue-white LED light with eight different combinations of photoperiod and PPFD, resulting four different daily light integrals (DLI). The DLI was increased by increasing PPFD (range 167 – 292 µmol m^-2^ s^-1^) at a constant photoperiod of 18 h (blue line) or by increasing photoperiod (range 12 – 21 hours) at a constant PPFD of 250 µmol m^-2^ s^-1^ (orange line). Purple diamond represents a third treatment at highest DLI, combining 350 µmol m^-2^ s^-1^ with 15 h photoperiod. Green line represents one common relationship with DLI, not different between DLI increase obtained by increased PPFD or by increased photoperiod. Asterisks indicate significant differences within the same DLI according to Fisher's protected LSD test at P = 0.05.*





***Figure S6.*** *Total chlorophyll of pak choi cultivars ‘Hybrid Special’ (A), ‘Shanghai Green’ (B), and ‘Red Summer’ (C) after four weeks of cultivation under red-blue-white LED light with eight different combinations of photoperiod and PPFD, resulting four different daily light integrals (DLI). The DLI was increased by increasing PPFD (range 167 – 292 µmol m^-2^ s^-1^) at a constant photoperiod of 18 h (blue line) or by increasing photoperiod (range 12 – 21 hours) at a constant PPFD of 250 µmol m^-2^ s^-1^ (orange line). Purple diamond represents a third treatment at highest DLI, combining 350 µmol m^-2^ s^-1^ with 15 h photoperiod. Green line represents one common relationship with DLI, not different between DLI increase obtained by increased PPFD or by increased photoperiod. Asterisks indicate significant differences within the same DLI according to Fisher's protected LSD test at P = 0.05.*





***Figure S7.*** *Carotenoid of pak choi cultivars ‘Hybrid Special’ (A), ‘Shanghai Green’ (B), and ‘Red Summer’ (C) after four weeks of cultivation under red-blue-white LED light with eight different combinations of photoperiod and PPFD, resulting four different daily light integrals (DLI). The DLI was increased by increasing PPFD (range 167 – 292 µmol m^-2^ s^-1^) at a constant photoperiod of 18 h (blue line) or by increasing photoperiod (range 12 – 21 hours) at a constant PPFD of 250 µmol m^-2^ s^-1^ (orange line). Purple diamond represents a third treatment at highest DLI, combining 350 µmol m^-2^ s^-1^ with 15 h photoperiod. Green line represents one common relationship with DLI, not different between DLI increase obtained by increased PPFD or by increased photoperiod. Asterisks indicate significant differences within the same DLI according to Fisher's protected LSD test at P = 0.05.*


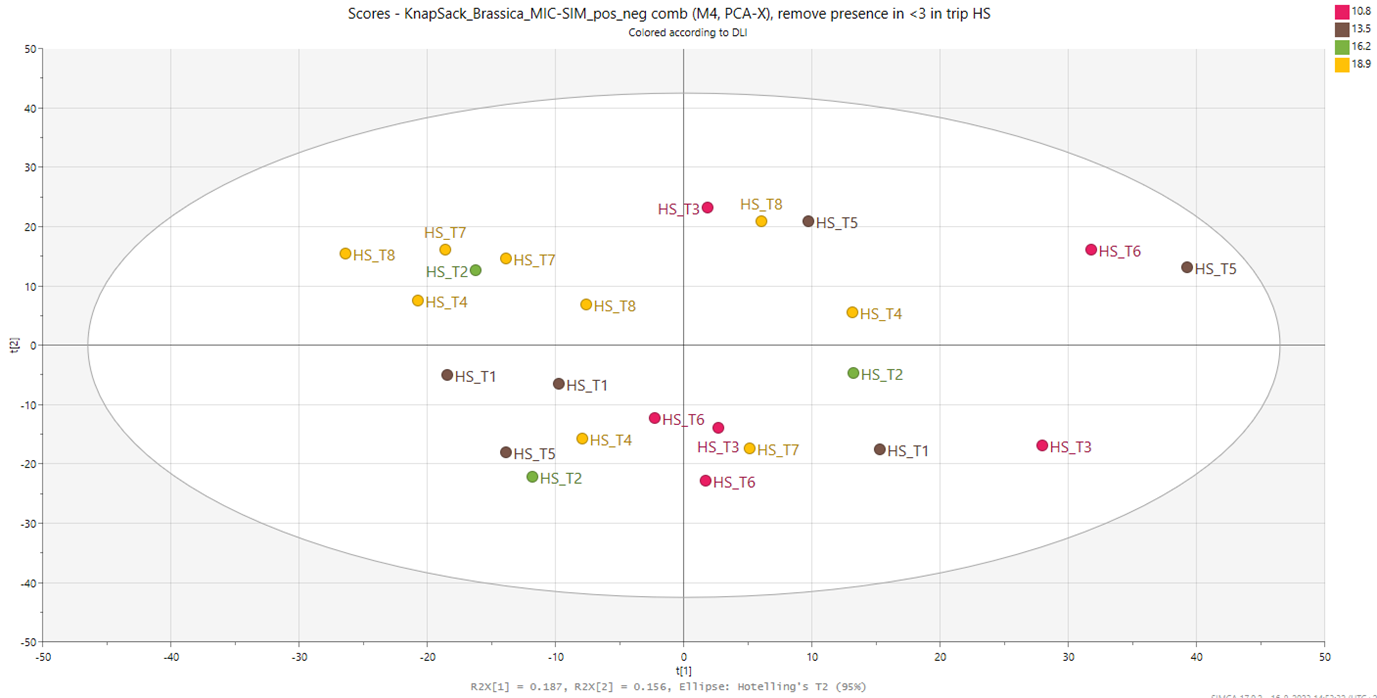


***Figure S8.*** *PCA of pak choi cultivar ‘Hybrid Special’ (HS) samples based on the variation in relative abundance of 1,591 compounds detected by the untargeted LCMS-based metabolomics approach. Plants were grown for four weeks under red-blue-white LED light with eight different combinations of photoperiod and light intensity, resulting four different daily light integrals (DLI). The circles enclose the four groups of samples exposed to the same DLI: 10.8 mol m^-2^ d^-1^ (red), 13.5 mol m^-2^ d^-1^ (brown), 16.2 mol m^-2^ d^-1^ (green) and 18.9 mol m^-2^ d^-1^ (yellow).*


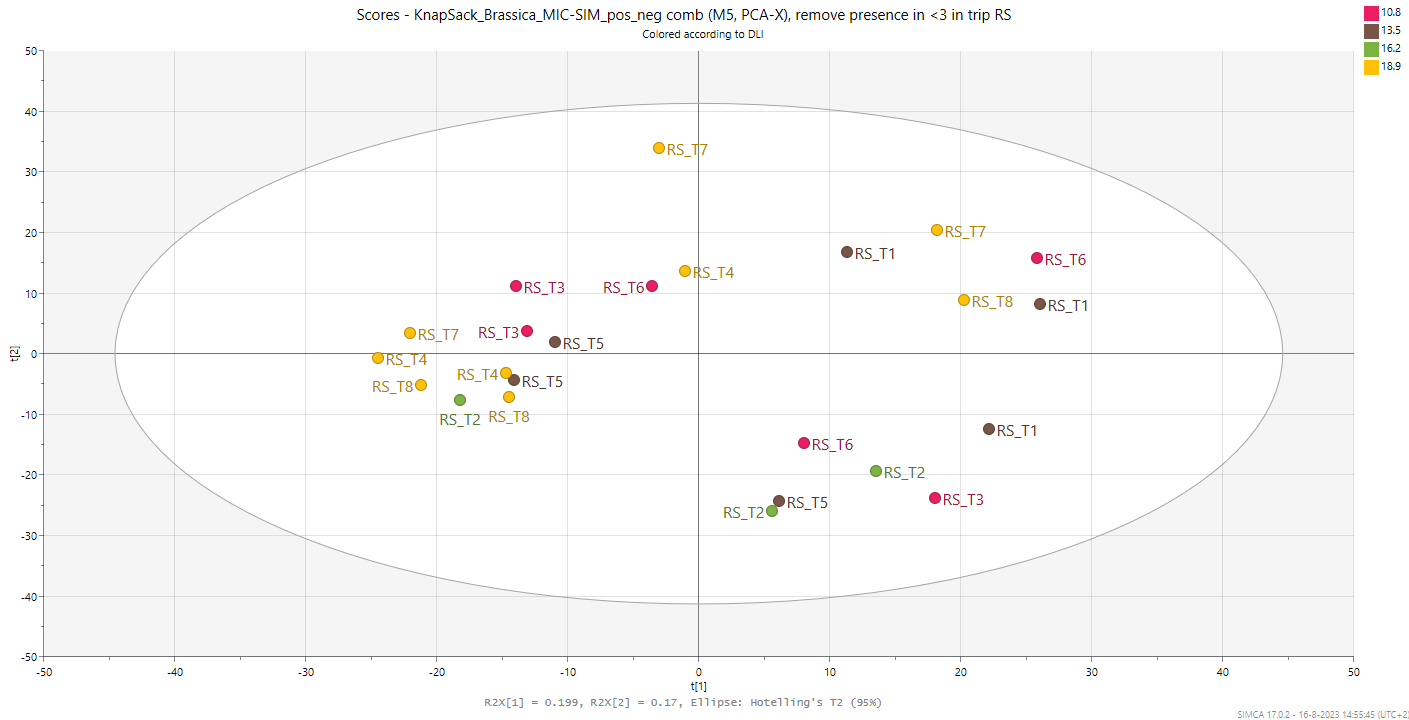


***Figure S9.*** *PCA of pak choi cultivar ‘Red Summer’ (RS) samples based on the variation in relative abundance of 1,591 compounds detected by the untargeted LCMS-based metabolomics approach. Plants were grown for four weeks under red-blue-white LED light with eight different combinations of photoperiod and light intensity, resulting four different daily light integrals (DLI).* *The circles enclose the four groups of samples exposed to the same DLI: 10.8 mol m^-2^ d^-1^ (red), 13.5 mol m^-2^ d^-1^ (brown), 16.2 mol m^-2^ d^-1^ (green) and 18.9 mol m^-2^ d^-1^ (yellow).*


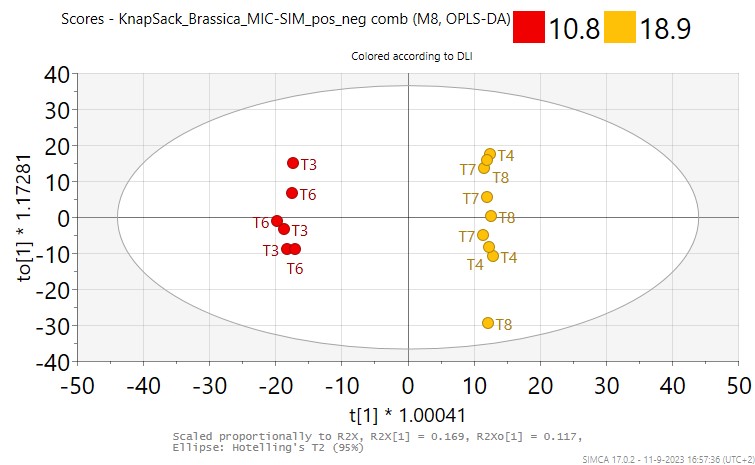


***Figure S10.*** *Supervised OPLS-DA plots of LC-MS data (+) for 'Shanghai Green' pak choi (SG) samples showed a separation between low DLI treatments (10.8 mol m⁻² d⁻¹, marked in red) and high DLI treatments (18.9 mol m⁻² d⁻¹, marked in yellow), indicating significant metabolic differences. Low DLI conditions include treatments T3 (PP 18 h, PPFD 167 µmol m⁻² s⁻¹) and T6 (PP 12 h, PPFD 250 µmol m⁻² s⁻¹), while high DLI include treatments T4 (PP 18 h, PPFD 292 µmol m⁻² s⁻¹), T7 (PP 21 h, PPFD 250 µmol m⁻² s⁻¹), and T8 (PP 15 h, PPFD 350 µmol m⁻² s⁻¹).*





***Figure S11.*** *Log-transformed values of untargeted metabolites across all three biological replicates in pak choi cultivar ‘Shanghai Green’ as a function of daily light integral (DLI) determined after four weeks of cultivation under red-blue-white LED light. DLI either increased by increasing PPFD (range 167 – 292 µmol m^-2^ s^-1^) at a constant photoperiod of 18 h (blue symbol) or by increasing photoperiod (range 12 – 21 hours) at a constant PPFD of 250 µmol m^-2^ s^-1^ (orange symbol). Purple diamond represents a third treatment at highest DLI, combining 350 µmol m^-2^ s^-1^ with 15 h photoperiod. Green line connects average values for each DLI.*

**Table S1**. Nutrient solution composition

| **Macronutrients** | **Concentration (mM)** |
| --- | --- |
| NH₄^⁺^ | 0.38 |
| K⁺ | 8.82 |
| Ca^₂⁺^ | 4.22 |
| Mg^₂⁺^ | 1.15 |
| NO₃⁻ | 12.92 |
| Cl⁻ | 1.53 |
| SO₄^2-^ | 1.53 |
| H2PO₄⁻ | 1.53 |
| Si | 0.38 |
| **Micronutrients** | **Concentration (mM)** |
| Mn | 3.8 |
| Zn | 3.8 |
| B | 38.3 |
| Cu | 0.77 |
| Mo | 0.38 |
| Fe | 30.07 |

**Table S2.** Overall visual quality description

| **OVQ score** | **Description of postharvest appearance** |
| --- | --- |
| **5** | Leaves are tender and shiny, with no decay, no wilting, no curly, do not show dark spots, and original color (green/red); stems are crisp. |
| **4** | Leaf slightly curls, initial wilting, initial tipburn, slight loss of initial leaf shininess, do not show dark spots + color change; stems are a slight loss of initial crispness. |
| **3** | Indicates the end of shelf-life. Leaves are slightly decayed, moderately shiny, with some wilting signs, slightly curly, some tipburn, show some dark spot presence, slightly color change; stems are moderately crisp. |
| **2** | Leaves are moderate decay, wilting, and curly, obvious, and widespread overall dark spot (< 50% total leaf area) presence, moderate color change; stems are overall loss of crispness. |
| **1** | Leaves show no leaf shininess, completely decayed, completely wilted, heavy curly leaf, obvious and widespread (>50% total leaf area) overall dark spot presence, overall color change; stems are completely loss of crispness. |

**Table S4.** Regression Equations Derived from ANCOVA Analysis

| **Variables** | **Hybrid Special** | **Shanghai Green** | **Red Summer** |
| --- | --- | --- | --- |
| Fresh weight | PP 18 h:  *y* = 24.3 + 1.18 *x*  PPFD 250 µmol m^-2^ s-^1^:  *y* = 2.2 + 2.55 *x* | *y* = 1.09 + 2.58 *x* | PP 18 h:  *y* = -2.65 + 1.92 *x*  PPFD 250 µmol m^-2^ s-^1^: *y* = -20.6 + 3.15 *x* |
| Dry weight | PP 18 h:  *y* = 0.543 + 0.165 *x*  PPFD 250 µmol m^-2^ s-^1^:  *y* = -0.859 + 0.252 *x* | *y* = -0.9 + 0.239 *x* | PP 18 h:  *y* = -0.187 + 0.119 *x*  PPFD 250 µmol m^-2^ s-^1^:  *y* = -1.5 + 0.209 *x* |
| Dry matter content | *y* = 4.35 + 0.185 *x* | *y* = 4.23 + 0.163 *x* | *y* = 5.51 + 0.0366 *x* |
| Leaf area | *y* = 469 + 8.19 *x* | *y* = 290 + 17.3 *x* | PP 18 h:  *y* = 170 + 17.8 *x*  PPFD 250 µmol m^-2^ s-^1^:  *y* = -48.8 + 31.9 *x* |
| Specific leaf area | PP 18 h:  *y* = 355 – 9.82 *x*  PPFD 250 µmol m^-2^ s-^1^:  *y* = 435 – 14.9 *x* | PP 18 h:  *y* = 386 – 11.4 *x*  PPFD 250 µmol m^-2^ s-^1^: *y* = 423 – 13.6 *x* | PP 18 h:  *y* = 427 – 9.34 *x*  PPFD 250 µmol m^-2^ s-^1^:  *y* = 537 – 16.6 *x* |
| Photosynthesis rate | PP 18 h:  *y* = -0.683 + 0.584 *x*  PPFD 250 µmol m^-2^ s-^1^:  *y* = 9.6 – 0.0463 *x* | PP 18 h :  *y* = -1.72 + 0.667 *x*  PPFD 250 µmol m^-2^ s-^1^: *y* = 9.46 – 0.0393 *x* | PP 18 h: *y* = -0.139 + 0.344 *x*  PPFD 250 µmol m^-2^ s-^1^:  *y* = 7.34 – 0.117 *x* |
| Quantum yield of CO_2_ assimilation | *y* = 0.0536 + 4.95 × 10^-5^ *x* | PP 18 h:  *y* = 0.0445 + 6.75 × 10^-4^ *x*  PPFD 250 µmol m^-2^ s-^1^:  *y* = 0.0607 – 0.000396 *x* | PP 18 h:  *y* = 0.0327 – 2.04 × 10^-5^ *x*  PPFD 250 µmol m^-2^ s-^1^:  *y* = 0.0386 – 0.000387 *x* |
| Light Use Efficiency | PP 18 h:  *y* = 7.22 – 0.181 *x*  PPFD 250 µmol m^-2^ s-^1^:  *y* = 4.49 – 0.0162 *x* | *y* = 4.34 – 0.011 *x* | PP 18 h:  *y* = 2.41 + 0.0208 *x*  PPFD 250 µmol m^-2^ s-^1^:  *y* = 0.464 + 0.149 *x* |
| Energy Use Efficienc*y* | PP 18 h:  *y* = 85.9 – 2.16 *x*  PPFD 250 µmol m^-2^ s-^1^:  *y* = 53.5 – 0.193 *x* | *y* = 51.7 – 0.131 *x* | PP 18 h:  *y* = 28.7 + 0.247 *x*  PPFD 250 µmol m^-2^ s-^1^:  *y* = 5.52 + 1.78 *x* |
| Vitamin C | *y* = 0.41 + 0.0302 *x* | *y* = 0.308 + 0.0375 *x* | *y* = 0.272 + 0.028 *x* |
| Total soluble sugar | *y* = -2.05 + 0.385 *x* | *y* = -0.754 + 0.322 *x* | *y* = 0.739 + 0.0312 *x* |
| Scavenging activity | *y* = 21.7 + 0.996 *x* | *y* = -91.1 + 18.1 *x* – 0.564 *x*^2^ | *y* = 42.4 + 0.571 *x* |
| Total chlorophyll | *y* = 0.263 + 0.0185 *x* | *y* = 0.35 + 0.00732 *x* | *y* = 0.291 + 0.0183 *x* |
| Carotenoid | *y* = 0.00679 + 0.0011 *x* | *y* = 0.00899 + 0.000753 *x* | *y* = 0.00357 + 0.00138 *x* |
| Cyanidin conjugate, pos_667 | *y* = 6.19 – 0.00858 *x* | *y* = 6.16 – 0.00542 *x* | PP 18 h:  *y* = 8.36 – 0.00757 *x*  PPFD 250 µmol m^-2^ s-^1^:  *y* = 8.1 + 0.00925 *x* |
| Glucobrassicanapin, neg_GSL7 | *y* = 9.32 + 0.0259 *x* | *y* = 8.8 + 0.0435 *x* | *y* = 8.88 + 0.0414 *x* |
| Glucobrassicin, neg_GSL8 | *y* = 7.88 + 0.00791 *x* | *y* = 8.14 + 0.0122 *x* | *y* = 7.73 + 0.0224 *x* |
| Pos_31 |  | *y* = 9.65 – 0.299 *x* |  |
| Pos_70 |  | *y* = 4.45 + 0.101 *x* |  |
| Neg_87 |  | *y* = 7.64 – 0.196 *x* |  |
| Neg_570 |  | *y* = 5.06 + 0.0755 *x* |  |

**Calculation for metabolites**

Table S5 shows the number of compounds with high positive correlations (>0.95) or high negative correlations (<-0.95) that appeared in at least one round for each cultivar.

**Table S5.** Calculation for high correlation

| **Cultivar** | **> 0.95** | **< -0.95** |
| --- | --- | --- |
| Hybrid Special | 11 | 2 |
| Red Summer | 2 | 1 |
| Shanghai Green | 32 | 3 |
| TOTAL | 45 | 6 |

Table S6 shows the number of compounds with positive correlations (>0.9, >0.8, and >0.7) or negative correlations (<-0.9, <-0.8, and <-0.7) across all three rounds for each cultivar. (color for easier readability)

**Table S6.** Calculation for all 3 rounds

| **Cultivar** | **> 0.9** | **< -0.9** | **> 0.8** | **< -0.8** | **> 0.7** | **< -0.7** |
| --- | --- | --- | --- | --- | --- | --- |
| Hybrid Special | 0 | 0 | 4 | 2 | 37 | 3 |
| Red Summer | 0 | 0 | 2 | 0 | 5 | 1 |
| Shanghai Green | 0 | 0 | 20 | 3 | 79 | 4 |
| TOTAL | 0 | 0 | 26 | 5 | 121 | 8 |

Table S7 shows the number of compounds where, in 2 rounds, the correlation are more than 0.90 and, in one round, the correlation is more than 0.70 (pos 0.9_0.7) as well as compounds where, in two rounds, the correlation is less than -0.90 and, in one round, the correlation is less than -0.70 (neg 0.9_0.7) for each cultivar.

**Table S7.** Calculation of 0.9-0.7

| **Cultivar** | **pos 0.9_0.7** | **neg 0.9_0.7** |
| --- | --- | --- |
| Hybrid Special | 5 | 1 |
| Red Summer | 0 | 0 |
| Shanghai Green | 14 | 1 |
| TOTAL | 19 | 2 |
